# Role of Cysteine Residues in the Structure, Stability, and Alkane Producing Activity of Cyanobacterial Aldehyde Deformylating Oxygenase

**DOI:** 10.1371/journal.pone.0122217

**Published:** 2015-04-02

**Authors:** Yuuki Hayashi, Fumitaka Yasugi, Munehito Arai

**Affiliations:** 1 Department of Life Sciences, Graduate School of Arts and Sciences, The University of Tokyo, Meguro, Tokyo, Japan; 2 PRESTO, Japan Science and Technology Agency, Kawaguchi, Saitama, Japan; MRC National Institute for Medical Research, UNITED KINGDOM

## Abstract

Aldehyde deformylating oxygenase (AD) is a key enzyme for alkane biosynthesis in cyanobacteria, and it can be used as a catalyst for alkane production *in vitro* and *in vivo*. However, three free Cys residues in AD may impair its catalytic activity by undesired disulfide bond formation and oxidation. To develop Cys-deficient mutants of AD, we examined the roles of the Cys residues in the structure, stability, and alkane producing activity of AD from *Nostoc punctiforme* PCC 73102 by systematic Cys-to-Ala/Ser mutagenesis. The C71A/S mutations reduced the hydrocarbon producing activity of AD and facilitated the formation of a dimer, indicating that the conserved Cys71, which is located in close proximity to the substrate-binding site, plays crucial roles in maintaining the activity, structure, and stability of AD. On the other hand, mutations at Cys107 and Cys117 did not affect the hydrocarbon producing activity of AD. Therefore, we propose that the C107A/C117A double mutant is preferable to wild type AD for alkane production and that the double mutant may be used as a pseudo-wild type protein for further improvement of the alkane producing activity of AD.

## Introduction

Alkanes are dominant moieties in crude petroleum and they are used as fuels for automobiles, jet planes, and power plants. However, worldwide consumption of petroleum not only depletes fossil fuel reserves but also causes global warming by releasing carbon dioxide. Therefore, it is desirable to develop sustainable resources that can function as substitutes for petroleum-based alkanes. Some plants and microorganisms produce alkanes *in vivo* [1–4]; therefore, biosynthesis of alkanes might be an alternative method for producing alkanes. Cyanobacteria can produce straight-chain alkanes/alkenes from fatty acyl-acyl carrier protein (fatty acyl-ACP), which is a metabolite in the fatty acid production pathway, using two enzymes, acyl-ACP reductase (AAR) and aldehyde deformylating oxygenase (also known as aldehyde decarbonylase; AD) [5]. AAR reduces fatty acyl-ACP to fatty aldehyde, which, in turn, is reduced to alkanes/alkenes by AD. Remarkably, *Escherichia coli* cells expressing both AAR and AD can produce and secrete C15 to C17 alkanes/alkenes [5], indicating the possibility of industrial biosynthesis of hydrocarbons using engineered microorganisms [5–8]. Alternatively, addition of AD protein to a solution of fatty aldehydes might be a promising method to produce hydrocarbons *in vitro*.

An industrial enzyme needs to be easy to handle with low production costs. Therefore, the presence of free Cys residues in an enzyme is problematic, especially when it is used *in vitro*, because improperly formed disulfide bonds may cause misfolding and aggregation and consequently impair the enzyme’s functional activity [9]. Even *in vivo*, overexpression of Cys-containing proteins in the cytoplasm may result in the formation of inclusion bodies [10,11]. In addition, oxidation of sulfur atoms in Cys residues may affect the activity and stability of the enzyme [12]. To avoid undesirable disulfide bond formation and oxidation, reducing agents such as dithiothreitol (DTT) and 2-mercaptoethanol are added to the enzyme solution; however, this process incurs additional costs. Therefore, it is desirable to decrease the number of free Cys residues in an enzyme by creating Cys-deficient variants without loss of catalytic activity [13,14]. However, free Cys residues play crucial roles in catalysis, as in the case of cysteine proteases [15], and thus substitution or deletion of free Cys residues in an enzyme may impair its function and stability. Therefore, it is necessary to investigate the effects of Cys substitutions on the activity, structure, and stability of an enzyme, prior to designing Cys-deficient mutants.

The free Cys residues in AD may impair its catalytic activity by undesired disulfide bond formation and oxidation. The functional mechanism of AD has been studied previously [3,4,16–21], and the crystal structure of AD from *Prochlorococcus marinus* MIT9313 (*Pm*AD) has been determined [22]. AD from *Nostoc punctiforme* PCC 73102 (*Np*AD) has higher hydrocarbon producing activity than ADs from other cyanobacterial strains [5]. *Np*AD does not have any disulfide bonds, but has three free Cys residues at positions 71, 107, and 117. However, the roles of these Cys residues are unknown. Here, we studied the effects of systematic and combinatorial Cys-to-Ala/Ser substitutions on the hydrocarbon producing activity, overall structure, and stability of *Np*AD. Our results revealed that the conserved Cys71 residue, which is located in close proximity to the substrate binding site, plays crucial roles in maintaining the activity, structure, and stability of AD. On the other hand, both the C107A and C117A mutations did not affect the hydrocarbon producing activity of AD, suggesting that a C107A/C117A double mutant of AD is preferable to wild type AD for alkane production.

## Materials and Methods

### Construction of plasmids

A DNA fragment coding for *Np*AD was prepared by overlap extension polymerase chain reaction (PCR). The codons were optimized for high-level expression in *E*. *coli* [23]. For the overexpression of AD, the *Np*AD DNA fragment was inserted into the pET-15b vector (Novagen) at the *Nde*I and *Bam*HI restriction sites, yielding the plasmid pET-15b-AD. For the coexpression of AD and AAR, the DNA fragment of *Np*AD was inserted into the pETDuet-1 coexpression vector (Novagen) at the *Nde*I and *Avr*II restriction sites, and subsequently the codon-optimized gene of AAR from *Synechococcus elongatus* PCC 7942, prepared by overlap extension PCR, was inserted into the same vector at the *Nco*I and *Eco*RI restriction sites, yielding the plasmid pRD. Both AD and AAR had a C-terminal extension (Gly-Ser-Ser-Gly) and a 6×His-tag to facilitate protein purification. All Cys-to-Ala/Ser mutants were created according to the method described in the QuikChange site-directed mutagenesis kit (Agilent Technologies, Santa Clara, CA, USA), with modifications.

### Measurement of hydrocarbon producing activity

*E*. *coli* BL21(DE3)pLysS competent cells were transformed with the pETDuet-1 coexpression vector containing both *AAR* and *AD*, inoculated onto a 2× YT agarose plate containing carbenicillin and chloramphenicol, and incubated at 37°C overnight. In a control experiment, an empty pETDuet-1 vector (without *AAR* or *AD*) was used. The colonies on the plate were seeded into 1 mL of M9 medium containing ampicillin and chloramphenicol, and precultured at 37°C overnight. The preculture was then seeded (at an optical density at 600 nm (OD_600_) of 0.1) into 3.1 mL of M9 medium containing 100 μM ammonium iron (II) sulfate and 1 mM isopropyl thiogalactoside (IPTG). The culture was aliquoted into a 96 deep well plate, with 1 mL in each well (3 wells per sample). The plate was incubated at 37°C for 16 h using a deep well plate incubator (Maximizer; TAITEC, Tokyo, Japan). A total of 1.5 mL of cell culture (0.5 mL from each well) was collected and sonicated for 5 min at intervals of 30 s using a Bioruptor Ultrasonicator UCD-250 (Cosmo-Bio, Tokyo, Japan). Five-hundred microliters of the cell lysate was centrifuged to separate the supernatant and pellet fractions, which were then used to quantify the amounts of soluble and insoluble forms of AAR and AD proteins by sodium dodecyl sulfate-polyacrylamide gel electrophoresis (SDS-PAGE) using a Gel Doc EZ system (BioRad). Eight-hundred microliters of the cell lysate was mixed with 800 μL of ethyl acetate by vortexing for 10 min in a glass vial. The organic phase was separated from the aqueous phase by centrifugation at 5,720 ×*g* for 10 min, and was replaced in another glass vial. The amounts of hydrocarbons (pentadecane (C15:0), heptadecene (C17:1), and heptadecane (C17:0)) produced in the cell culture were quantified by gas chromatography-mass spectrometry (GC-MS). The total amount of hydrocarbons produced in the culture was normalized to the amount of soluble form of AD protein expressed in *E*. *coli*, as estimated by SDS-PAGE (see above). The total normalized amount of hydrocarbons was compared with that produced by wild type AD, and the ratio thus obtained was used as an index of hydrocarbon producing activity. All measurements were carried out in duplicate or quadruplicate.

GC-MS measurements were made using a Shimadzu gas chromatograph mass spectrometer GCMS-QP2010 Ultra (Shimadzu, Kyoto, Japan). One microliter of sample was injected into a 30 m Rxi-5ms capillary column (0.25 mm internal diameter) by splitless injection. The GC inlet temperature was 290°C. The oven temperature was maintained at 75°C for 5 min, ramped up to 290°C at a rate of 10°C/min, and then held at 290°C for an additional 9 min. The flow rate of the carrier gas helium was 1.3 mL/min. The GC-MS interface temperature was 290°C, and the ion source temperature was 230°C. The MS quadrapole scanned from 25 to 150 m/z. Retention times and fragmentation patterns of product peaks were compared with those of alkane standard solution C_8_-C_20_ (Sigma-Aldrich) to confirm peak identities.

### Western blotting

The amount of AAR coexpressed in *E*. *coli* with AD was quantified by western blotting, because the SDS-PAGE band corresponding to AAR overlaps with the thick band derived from an *E*. *coli* protein (~37 kDa). The supernatant and pellet fractions of the cell lysate were applied to SDS-PAGE. The proteins were electrotransferred onto a PVDF membrane (Immobilon-P, Millipore), which was then blocked with 100 mL of phosphate buffer saline (PBS) containing 5% skim milk at room temperature for 1 h with agitation. His-tagged AAR was detected with anti-His-tag antibody conjugated with horseradish peroxidase (Medical & Biological Laboratories, Japan) and 3,3',5,5'-tetramethylbenzidine (TMB) (ATTO, Japan).

### Protein expression and purification

*E*. *coli* BL21(DE3)pLysS cells, harboring the expression vector pET-15b-AD or its variants, was precultured in 10 mL of 2× YT medium containing ampicillin and chloramphenicol at 37°C overnight. The preculture was seeded into 1 L of 2× YT medium, and the culture was incubated in a shaker at 37°C. Protein expression was induced by the addition of 1 mM IPTG at OD_600_ of 0.6–0.9. After incubation for 4 h at 37°C, the culture was centrifuged at 12,310 ×*g* for 15 min at 4°C, and the resultant cell pellet was stored at—80°C. Then, the cells were resuspended with ~30 mL of wash buffer (50 mM sodium phosphate (pH 7.4), 300 mM NaCl, 10 mM imidazole, and 3 mM DTT), and the suspension was sonicated on ice for 4 min using a Branson Sonifier 250D (Central Scientific, Tokyo, Japan). The lysate was centrifuged at 35,140 ×*g* for 30 min at 4°C. The resultant supernatant was filtered with a membrane filter (pore size, 0.45 μm), and incubated with a Ni-agarose column (Wako Pure Chemical Industries, Osaka, Japan) at room temperature for 15 min. The column was washed with wash buffer and incubated with 20 mL of elution buffer (50 mM sodium phosphate (pH 7.4), 300 mM NaCl, 500 mM imidazole, and 3 mM DTT) before elution of AD protein. The eluate was desalted by dialysis in buffer B (10 mM sodium phosphate (pH 7.2), 150 mM NaCl, and 1 mM DTT).

### Size exclusion chromatography

Size exclusion chromatography was performed using an ÄKTA explorer 10S chromatography system (GE Healthcare). Five-hundred microliters of the protein solution, adjusted to 2.5 mg/mL by dilution or concentration with an Amicon Ultra-4 centrifugal filter, was applied onto a Superdex 200pg column with a column volume of 124 mL (GE Healthcare) at a flow rate of 0.5 mL/min in running buffer (10mM sodium phosphate (pH 7.2) and 150 mM NaCl). The calibration standard was prepared using lysozyme (14 kDa, Wako), carbonic anhydrase (29 kDa, Sigma-Aldrich), ovalbumin (44 kDa, Sigma-Aldrich), bovine serum albumin (66 kDa, Nacalai Tesque), and alcohol dehydrogenase (150 kDa, Oriental Yeast Co., Ltd.).

### Circular dichroism (CD) spectra

CD spectra were measured in a JASCO J-805 spectropolarimeter (JASCO, Tokyo, Japan) at 200–250 nm with a quartz cuvette of 1 mm path length at 25°C (controlled by a thermostat circulating water bath). The monomer fractions of the AD variants obtained by size exclusion chromatography were used for the measurements. Protein concentration was 0.2 mg/mL, as determined spectrophotometrically (the extinction coefficient at 280 nm was assumed to be 22,920 M^-1^ cm^-1^) [24]. Mean residue ellipticity was calculated as described previously [25].

### Thermal denaturation

Thermal denaturation of AD was monitored by the ellipticity at 222 nm using a JASCO J-805 spectropolarimeter with a quartz cuvette of 1 mm path length. Protein concentration was 0.2 mg/mL. The temperature in the cuvette was controlled by a thermostat circulating water bath, calibrated by a thermocouple. The temperature was increased from 6 to 92°C at a rate of 1.2 ± 0.2°C/min. *T*_m_ was defined as the temperature at which the fraction of native state was 50%. *T*_m_ for second transition was defined as the temperature at which the intermediate and unfolded states existed in equal amounts. The thermal transition curve was normalized against the baseline values obtained for the native and unfolded states.

## Results

### Cys-deficient mutants of AD

*Np*AD has three free Cys residues at positions 71, 107, and 117. Multiple amino acid sequence alignment of the AD-family proteins in the UniProt database (133 sequences)[26] revealed that Cys71 and Cys107 are completely conserved, while the amino acid at position 117 is Ala (46%), Cys (42%), Ser (9%), or Thr (3%) (S1 Fig). Conservation of Cys71 and Cys107 suggests that these residues play important roles in the function, structure, and/or stability of AD.

**Fig 1:**
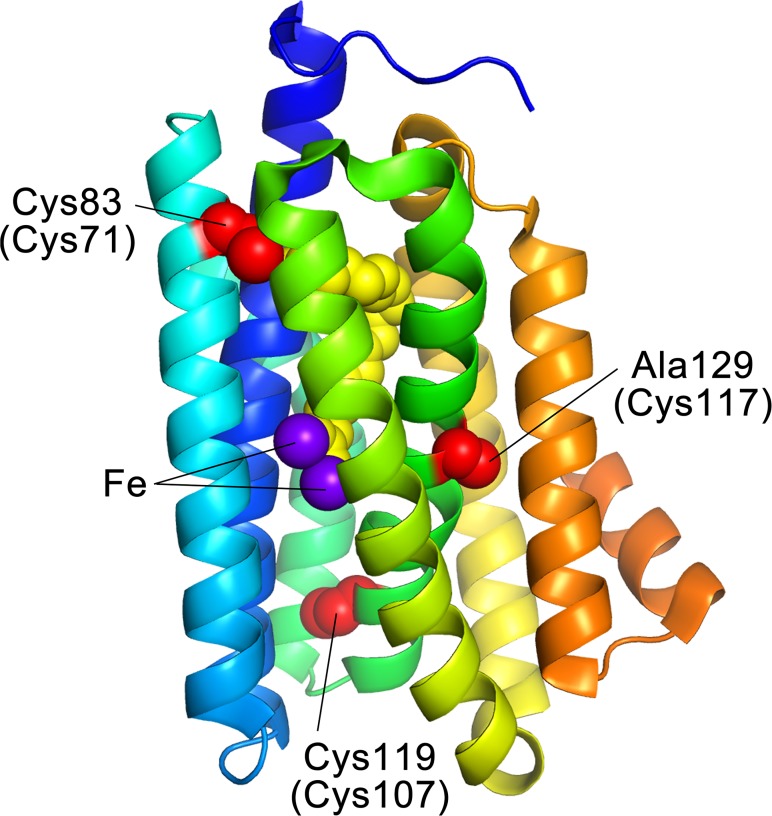
Crystal structure of *Prochlorococcus marinus* MIT9313 aldehyde deformylating oxygenase (*Pm*AD). Cys83, Cys119, and Ala129 in *Pm*AD, which corresponds to Cys71, Cys107, and Cys117 in AD from *Nostoc punctiforme* PCC 73102 (*Np*AD), respectively, are shown as red space-fill models (PDB ID: 2OC5). The two iron atoms and the substrate are shown as purple and yellow balls, respectively. The α-helices neighboring the helix involving Cys71 (cyan) are shown in blue and yellow-green. The figure was drawn using the PyMOL Molecular Graphics System, Schrödinger, LLC.

To examine the roles of the three free Cys residues, we introduced substitutions at these positions and compared the activity, structure, and stability of the mutants with that of wild type AD. Because of similarities in their side chains, a Cys residue is often substituted to Ala (hydrophobic) or Ser (hydrophilic), depending on the environment of the Cys residue in a protein, to create Cys-deficient mutant proteins [27]. The crystal structure of *Pm*AD revealed that Cys83, Cys119, and Ala129 in *Pm*AD, which correspond to Cys71, Cys107, and Cys117 in *Np*AD, respectively, are completely buried inside the protein molecule and are surrounded by a hydrophobic environment (Fig 1). Therefore, we initially replaced the Cys residues with Ala, and created the following single, double, and triple Cys-to-Ala mutants of *Np*AD: C71A, C107A, C117A, C71A/C107A, C71A/C117A, C107A/C117A, and C71A/C107A/C117A.

### Activity of the Cys-deficient mutants

**Fig 2:**
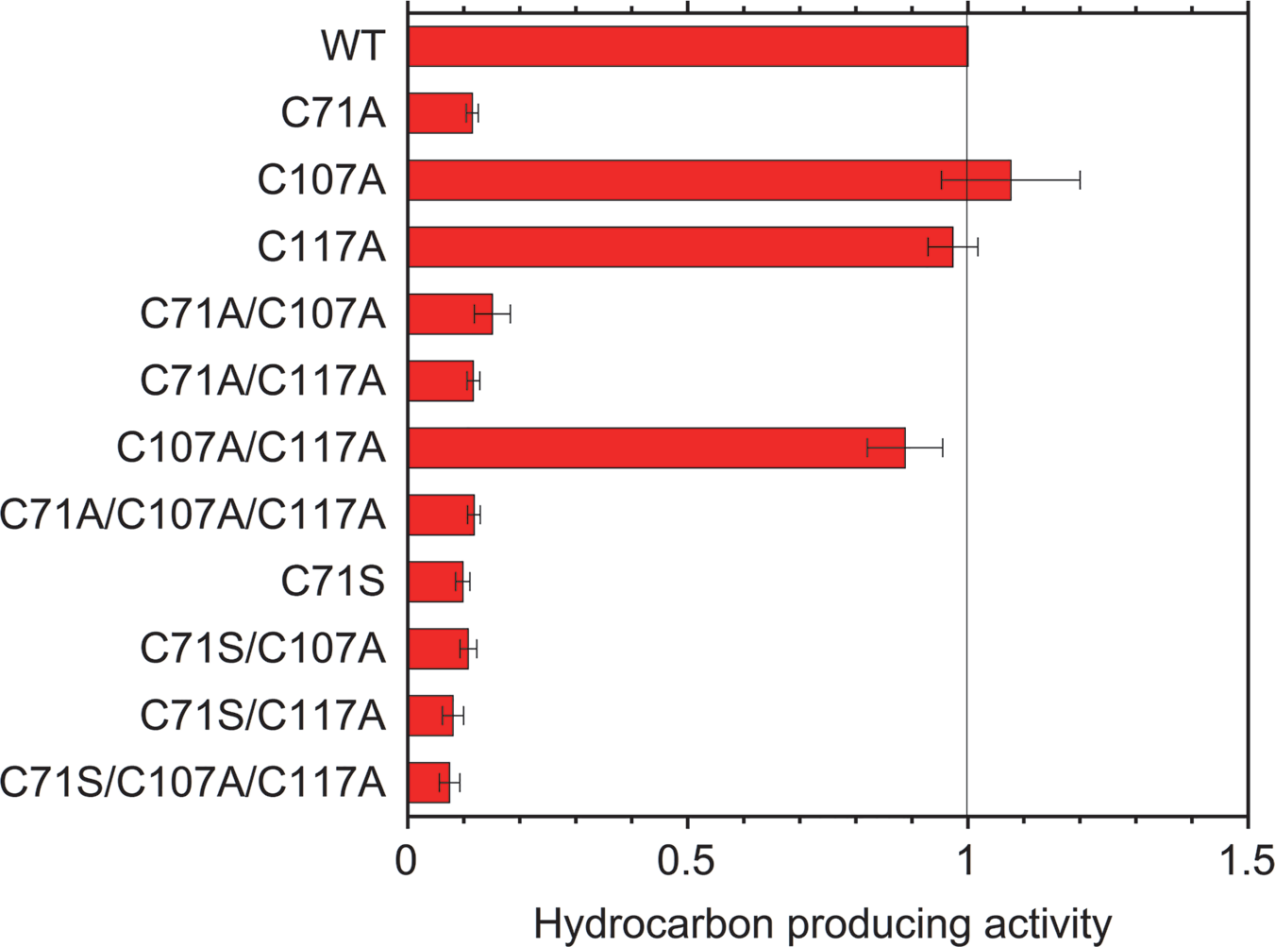
Hydrocarbon producing activities of the wild type and mutant ADs. The activity value presented here is relative to that of the wild type. The data are means ± standard deviations of duplicate or quadruplicate experiments.

The hydrocarbon producing activities of the wild type and mutant ADs were assessed by estimating the amount of hydrocarbons produced in *E*. *coli* cells coexpressing AD and AAR proteins (Fig 2 and Table 1). A GC-MS profile of the cell culture revealed the presence of high amounts of pentadecane and heptadecene and a small amount of heptadecane (S2A Fig). The hydrocarbon producing activity was calculated as the total amount of hydrocarbons produced, which was then normalized by the amount of soluble form of AD protein in *E*. *coli*, as quantified by SDS-PAGE (S3 Fig and S4 Fig). The amount of AAR coexpressed in *E*. *coli* with AD was unchanged by the mutations in AD (S5 Fig). In a control experiment, no hydrocarbons are detected in *E*. *coli* cell culture that did not express AAR or AD (S2B Fig). Michaelis parameters, *k*_cat_ and *K*_m_, were not determined, because the substrates of AD, hexadecanal and octadecanal, are highly insoluble under physiological conditions [17,21].

**Table 1:** Activity, stability, and structural properties of the wild type and mutant aldehyde deformylating oxygenases (ADs).

Protein	Activity^1^	Expression level^2^	Solubility (%)^3^	Fraction of dimers (%)^4^	*T*_m_ (°C)^5^
Wild type	1.00 ± 0.01^6^	1.00 ± 0.01	+	1.2 ± 0.2	53
C71A	0.12 ± 0.01	0.56 ± 0.05	+	7 ± 3	47
C107A	1.1 ± 0.1	0.89 ± 0.06	+	1.5 ± 0.4	47
C117A	0.97 ± 0.04	0.98 ± 0.07	+	1.5 ± 0.4	47
C71A/C107A	0.15 ± 0.03	0.52 ± 0.06	+/–	2.2 ± 0.8	48 (76)
C71A/C117A	0.12 ± 0.01	0.53 ± 0.03	+/–	1.3 ± 0.5	47 (78)
C107A/C117A	0.89 ± 0.07	0.92 ± 0.07	+/–	1.5 ± 0.6	43 (76)
C71A/C107A/C117A	0.12 ± 0.01	0.62 ± 0.02	–	n.d.^7^	n.d.
C71S	0.10 ± 0.01	0.60 ± 0.08	+/–	6 ± 1	46
C71S/C107A	0.11 ± 0.02	0.50 ± 0.04	n.d.	n.d.	n.d.
C71S/C117A	0.08 ± 0.02	0.61 ± 0.01	n.d.	n.d.	n.d.
C71S/C107A/C117A	0.07 ± 0.02	0.60 ± 0.03	–	n.d.	n.d.

^1^Hydrocarbon producing activity relative to that of the wild type.

^2^Expression level of AD protein, including the soluble and insoluble forms, in *Escherichia coli* is shown relative to that of the wild type.

^3^Solubility of AD when only AD was overexpressed in *E*. *coli* for *in vitro* characterization of the structure and stability. +: > 60% soluble; +/–: 20–60% soluble;–: < 20% soluble.

^4^Fraction of dimers (%), as estimated by size exclusion chromatography.

^5^Melting temperature, as measured by thermal denaturation. Errors are ±1°C. The *T*_m_ for the second transition is also shown for the double mutants in parenthesis.

^6^The means and standard deviations of duplicate or quadruplicate measurements are shown.

^7^not determined.

The C107A and C117A single mutants and the C107A/C117A double mutant had hydrocarbon producing activities comparable to that of the wild type (Fig 2). These results indicate that both C107A and C117A mutations do not affect the activity of AD.

On the other hand, the C71A mutation significantly reduced the hydrocarbon producing activity of AD. The activity of the C71A single mutant was only 0.1-fold that of wild type AD (Fig 2). The C71A/C107A and C71A/C117A double mutants and the C71A/C107A/C117A triple mutant also had low activities similar to the C71A single mutant. Remarkably, the expression level of the C71A-containing mutants was only ~60% that of the wild type (Table 1). The hydrocarbon producing activity shown in Fig 2 was calculated after taking into account the amount of soluble form of AD (see above). Therefore, the low amounts of hydrocarbons produced by the C71A-containing mutants are attributed to their low catalytic efficiencies.

Because the C71A mutation impaired hydrocarbon production, we substituted Cys71 into Ser. The C71S variants (C71S, C71S/C107A, C71S/C117A, and C71S/C107A/C117A) had activities comparable to or even lower than the activities of the C71A variants (Fig 2). This indicates the importance of Cys71 for the catalytic efficiency of AD.

### Effects on the stability of the iron atoms

Crystal structure of AD shows that residues interacting with two iron atoms are Glu33, Glu61, His64, Glu116, Glu145, and His148 in *Np*AD [22]. Although the Cys residues in AD do not directly interact with these residues, the mutations at Cys117, which is next to Glu116, may affect the stability of the iron atoms. To investigate the effects of Cys-to-Ala/Ser mutations on the stability of the iron atoms in AD, we measured the iron concentration dependence of the hydrocarbon producing activities of the wild type and mutant ADs. The results show that the amount of hydrocarbons produced in *E*. *coli* is decreased as the iron concentration in the M9 medium is decreased, and a large decrease in the amount of hydrocarbons is observed when the iron concentration is dropped from 10 to 1 μM (S6 Fig). Such behaviors are observed for all the mutants and the wild type, indicating that the Cys-to-Ala/Ser mutations do not affect the iron binding to AD.

### Structural features of the Cys-deficient mutants

To investigate the structure and stability of the Cys-to-Ala and C71S mutants of AD, we overexpressed them in *E*. *coli* and purified their soluble cell-lysate fractions by Ni-affinity chromatography and subsequently by size exclusion chromatography. Most of the proteins were soluble, but the C71A/C107A/C117A triple mutant formed inclusion bodies when overexpressed in *E*. *coli* (Table 1). Because denaturant-induced denaturation of AD was not reversible, we did not investigate the C71A/C107A/C117A triple mutant further.

**Fig 3:**
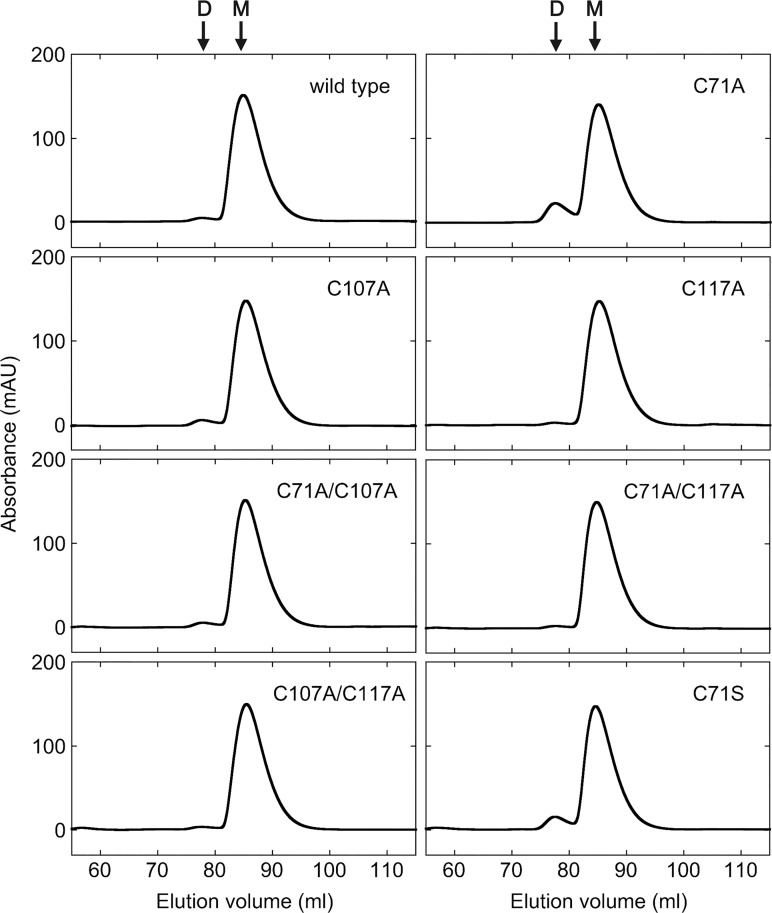
Size exclusion chromatography elution profiles of the wild type and mutant ADs. D and M denote the elution volumes of a dimer and a monomer, respectively.

Elution profiles obtained in size exclusion chromatography experiments revealed that the wild type and mutant ADs were predominantly in a monomeric state (Fig 3 and Table 1). However, the C71A and C71S single mutants formed dimers more than the wild type and other mutants. SDS-PAGE of the dimer fraction show that this fraction is composed of AD protein, confirming the formation of AD dimers.

To elucidate the mechanism underlying dimer formation in C71A, we purified the dimer fraction of C71A, treated it with 100 mM DTT, and subjected the sample to size exclusion chromatography using running buffer containing 5 mM DTT (S7 Fig). The results revealed that the DTT-treated dimer was still in a dimeric state, indicating that dimer formation in C71A and C71S is not caused by disulfide bond formation, but possibly by intermolecular hydrophobic interactions. AD proteins that were present in a monomeric state were used in the subsequent experiments.

**Fig 4:**
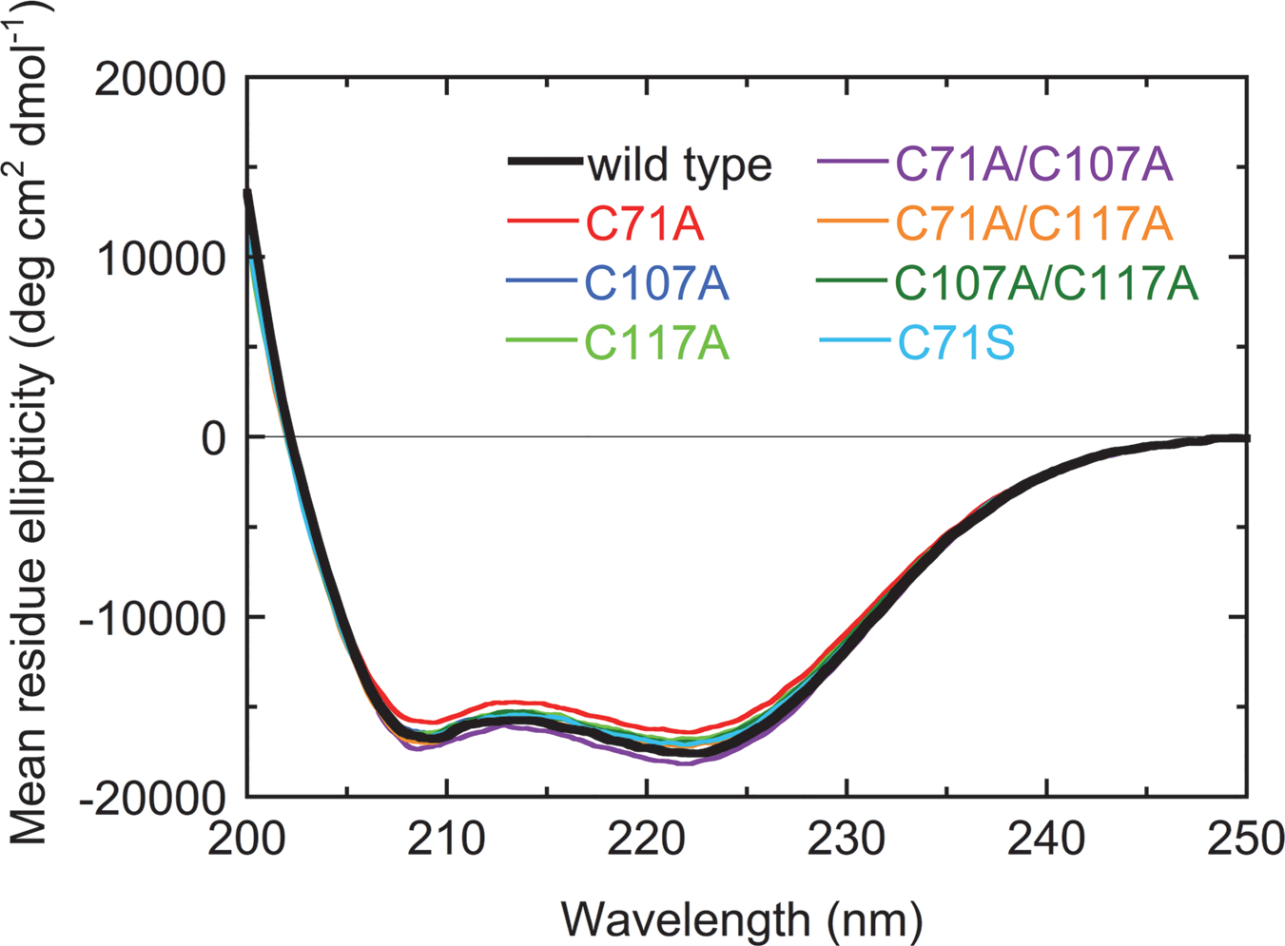
Far-ultra violet (UV) circular dichroism (CD) spectra of the wild type and mutant ADs.

The far-ultra violet (UV) CD spectra of the wild-type and mutant ADs revealed two minima at approximately 208 and 222 nm, typical of the CD spectra of proteins comprised mostly of α-helices (Fig 4). These results are consistent with the predominantly α-helical structure of *Pm*AD (Fig 1). The spectra obtained for the mutant ADs were similar to that of the wild type, except in the case of C71A and C71A/C107A, indicating that overall structure was not affected by the mutations. However, the CD ellipticities of the C71A and C71A/C107A mutants were slightly higher and lower than that of the wild type, respectively. This might indicate small perturbations in secondary structure by the mutations and/or reflect uncertainties in the determination of protein concentrations.

### Stability of the Cys-deficient mutants

**Fig 5:**
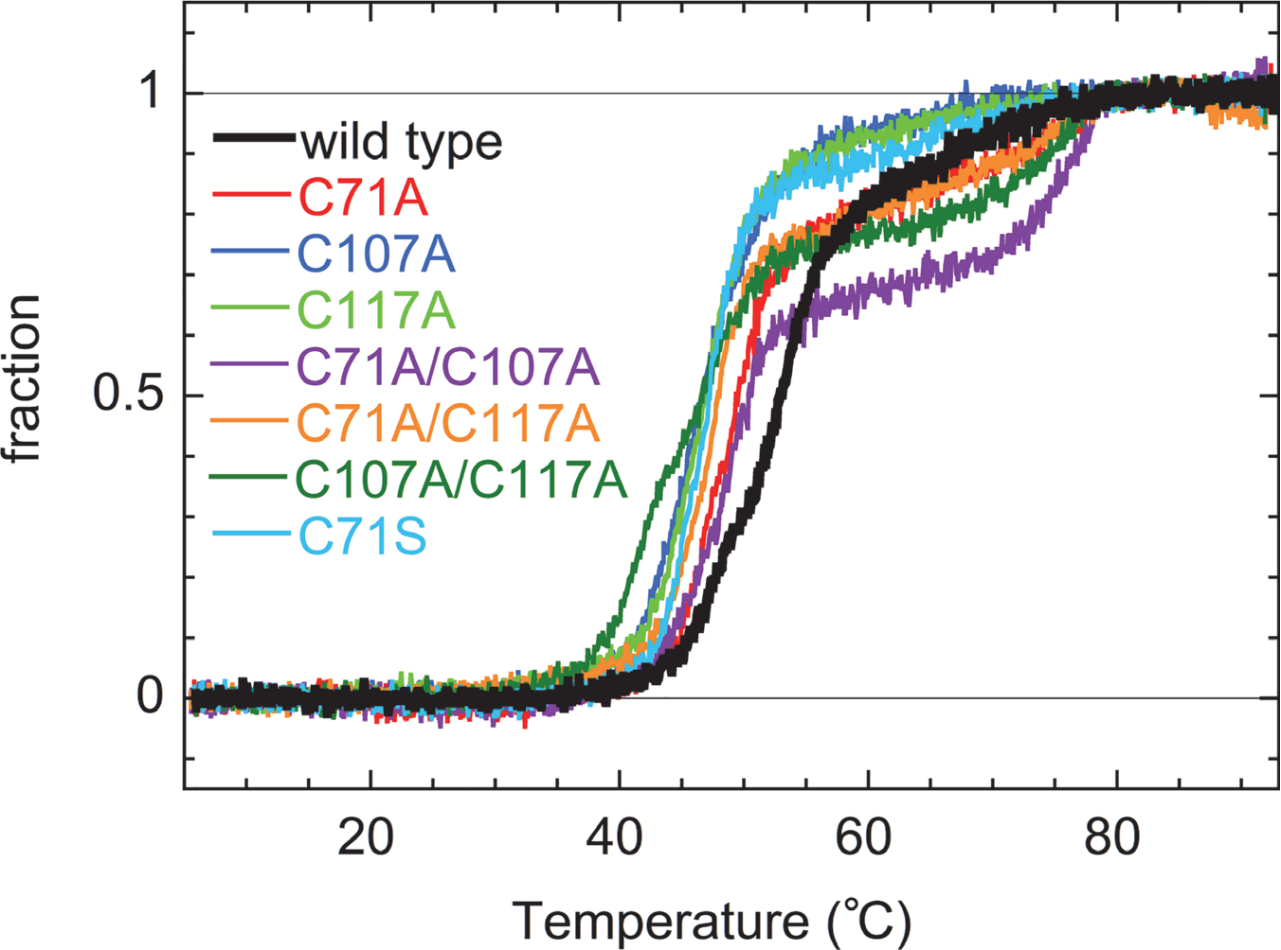
Thermal denaturation curves of the wild type and mutant ADs. The denaturation curves were monitored by the CD ellipticity at 222 nm (S8 Fig). The values were then normalized to the baseline values of the native and unfolded states. A two-step denaturation was observed for the double mutants.

Fig 5 shows the thermal denaturation curves of the wild type and mutant ADs. Wild type AD unfolded in a two-state manner with a melting temperature, *T*_m_, of 53°C (Table 1). Remarkably, all the single mutants had a *T*_m_ value 6–7°C lower than that of the wild type, indicating that the Cys-to-Ala/Ser mutations destabilize the AD protein. The C107A/C117A double mutant was more unstable than the C107A and C117A single mutants in a quasi-additive manner. Thus, it is possible that the C71A/C107A/C117A triple mutant was more destabilized than the double mutant, and it was partially denatured even at 37°C, leading to the formation of inclusion bodies when overexpressed in *E*. *coli*.

Interestingly, two-step transitions with a second *T*_m_ at 76–78°C were observed during the denaturation process of the double mutants (Fig 5 and Table 1). This indicates that combinations of the Cys-to-Ala mutations destabilized the native state but stabilized the intermediate state, resulting in the accumulation of the partially folded intermediate.

## Discussion

### Roles of Cys residues in AD

Table 1 summarizes the activity, stability, and structural properties of the wild type and mutant ADs. The C71A, C107A, and C117A mutations destabilize the protein, indicating that three free Cys residues play a role in stabilizing AD. Moreover, the C71A and C71S mutations significantly diminished the hydrocarbon producing activity of AD, and the mutants had a tendency to form dimers, indicating that the conserved Cys71 residue is indispensable for the function and structure of AD. The crystal structure of *Pm*AD suggests that Cys71 participates in forming the substrate-binding pocket and is located in close proximity to the substrate (Fig 1). Therefore, mutations at Cys71 may perturb the association and/or dissociation of the substrate, leading to lower hydrocarbon producing activity. The crystal structure also suggests that the dimerization tendency of the C71A and C71S mutants may be attributed to movement of the α-helices neighboring the helix involving Cys71 (Fig 1), thereby exposing the buried hydrophobic residues adjacent to Cys71, leading to the formation of a dimeric state by intermolecular hydrophobic interactions.

On the other hand, neither Cys107 nor Cys117 are in close proximity to the substrate or the two iron atoms that are considered part of the active site (Fig 1). This suggests that both Cys107 and Cys117 do not have any functional roles, consistent with the present results that the C107A and C117A mutations do not reduce the hydrocarbon producing activity. Thus, the conserved Cys107 residue of cyanobacterial ADs probably plays a role in maintaining the stability of AD, and not in the activity or the structure.

### Cys-deficient mutants of AD

Cys-deficient enzymes are preferable in industrial use, because they are easy to handle with low costs and are devoid of undesired disulfide bonds that may impair proper folding and function. Our results revealed that the C107A and C117A mutations did not affect the hydrocarbon producing activity. In contrast, the C71A and C71S mutations significantly decreased the hydrocarbon producing activity, and both mutants had a tendency to form dimers. Taken together, these results suggest that the C107A and C117A mutations are appropriate for the preparation of Cys-deficient AD proteins, while the C71A and C71S are not acceptable. Therefore, we propose that the C107A/C117A double mutant of AD, which had a hydrocarbon producing activity comparable to that of the wild type enzyme, is preferable to wild type AD for alkane production.

Although alkane biosynthesis using AAR and AD might be a promising method for the production of alkanes that may be used as alternatives to petroleum-based alkanes, the catalytic activities of both enzymes are very low [5,22]. Therefore, it is necessary to improve their activities before they can be used in biofuel production. We propose that the C107A/C117A double mutant is suitable as a pseudo-wild type enzyme for developing a highly active AD protein by directed evolution and protein engineering.

## Conclusions

By systematic Cys-to-Ala/Ser mutagenesis, we investigated the roles of three free Cys residues in *Np*AD. The conserved Cys71, which is located in close proximity to the substrate-binding site, plays crucial roles in maintaining the activity, structure, and stability of AD, probably by mediating the association and/or dissociation of the substrate. Thus, the C71A and C71S mutations lowered the hydrocarbon producing activity and had a tendency to form dimers. On the other hand, although Cys107 and Cys117 play a role in stabilizing the protein, they are not involved in catalysis for alkane production. Therefore, the C107A/C117A double mutant, which had activity comparable to that of the wild type, is preferable to wild type AD for alkane production and can be used as a pseudo-wild type enzyme for further improvement of the alkane producing activity of AD.

## Supporting Information

S1 Fig Multiple sequence alignment of the AD-family proteins. One hundred and thirty-three sequences in the UniProt database were used. Multiple sequence alignment was performed using Clustal Omega [28]. The amino acid sequence and the residue number of *Np*AD are shown in blue at the top. Cys residues located at positions 71, 107, and 117 of *Np*AD are shown in red.
(PDF)

S2 Fig Gas chromatography-mass spectrometry (GC-MS) profiles of the *Escherichia coli* cell cultures. (A) GC-MS profile of the *E*. *coli* cell culture coexpressing wild type AD and AAR proteins. The peaks after 19 min of the elution time were derived from *E*. *coli* cells. (B) GC-MS profile of the *E*. *coli* cell culture that did not express either AAR or AD (control). Peaks for pentadecane, heptadecene, and heptadecane, which should appear between 14–17 min, were not observed.
(TIF)

S3 Fig Sodium dodecyl sulfate-polyacrylamide gel electrophoresis (SDS-PAGE) of the supernatant and pellet fractions of *E*. *coli* cell cultures expressing the wild type and mutant ADs. The cell culture was sonicated and centrifuged to separate the supernatant (s) and pellet (p) fractions. Lane 1 is the molecular weight marker (M). The band for AD (28 kDa) is indicated by an arrow.
(TIF)

S4 Fig The amount of soluble form of the wild type and mutant ADs coexpressed with AAR in *E*. *coli*. The value was quantified by SDS-PAGE. Values relative to that of the wild type are shown. The data are means ± standard deviations of duplicate or quadruplicate experiments.
(TIF)

S5 Fig Western blotting of AAR coexpressed in *E*. *coli* with the wild type and mutant ADs. The cell culture was sonicated and centrifuged to separate the supernatant (s) and pellet (p) fractions. Lanes 1 and 13 in the upper and lower gels, respectively, are the molecular weight marker (M).
(TIF)

S6 Fig Iron concentration dependence of hydrocarbon producing activities of the wild type and mutant ADs. The amount of hydrocarbons produced in *E*. *coli*, which was cultured in the M9 medium containing 0, 1, 10, or 100 μM ammonium iron (II) sulfate, was normalized by the amount of soluble form of AD protein in *E*. *coli*, as estimated by SDS-PAGE. The activity value presented here is relative to that of the wild type in the presence of 100 μM ammonium iron (II) sulfate. The data are means ± standard deviations of duplicate or quadruplicate experiments.
(TIF)

S7 Fig Size exclusion chromatography elution profiles of the dimer fraction of C71A with or without DTT treatment. (Red) The dimer fraction of C71A was purified by size exclusion chromatography, concentrated with an Amicon Ultra-4 centrifugal filter, treated with 100 mM DTT at 4°C for 3 h, and subjected to size exclusion chromatography with running buffer containing 5 mM DTT. (Blue) The elution profile of the purified and concentrated dimer fraction of C71A applied to size exclusion chromatography using running buffer without DTT. (Black) The elution profile of wild type AD is shown as a control. The absorbance scales for C71A and wild type AD are shown at the left and right vertical axes, respectively. Here, a shorter column is used compared to that used in the experiments shown in Fig 3.
(TIF)

S8 Fig Un-normalized thermal denaturation curves of the wild type and mutant ADs. (TIF)
