# Supplementary material for: Role of Cysteine Residues in the Structure, Stability, and Alkane Producing Activity of Cyanobacterial Aldehyde Deformylating Oxygenase
Source: PLoS One. 2015 Apr 2;10(4):e0122217. doi: 10.1371/journal.pone.0122217 (PMC4383598; doi:10.1371/journal.pone.0122217)
Supplement: S1 Fig — One hundred and thirty-three sequences in the UniProt database were used. Multiple sequence alignment was performed using Clustal Omega [28]. The amino acid sequence and the residue number of NpAD are shown in blue at the top. Cys residues located at positions 71, 107, and 117 of NpAD are shown in red. (PDF) [file pone.0122217.s001.pdf]

```
                                                                 1.......10........20......
                                                                             : .  *:.:: *::
sp|B2J1M1|ALDEC_NOSP7      ----------------------------------MQQLTDQSKELDFKSETYKDAYSRIN
tr|U5QDK0|U5QDK0_9CYAN     ------------------------------MNRTDESTAAGAPALDYRSETYRDAYSRIN
tr|Q7NGM3|Q7NGM3_GLOVI     ------------------------------MN----RTAPSSAALDYRSDTYRDAYSRIN
tr|Q2JJ13|Q2JJ13_SYNJB     ---MNVLPNTPQ-----------------------PLADEGGTTLDYGSAVYRQAYSRIN
tr|Q2JX62|Q2JX62_SYNJA     MAPANVLPNTPP-----------------------SPTDGGGTALDYSSPRYRQAYSRIN
tr|K9RTZ6|K9RTZ6_SYNP3     -----------------------------------MSP-IQAPTLDYTSETYKDAYSRIN
tr|Q8DJB4|Q8DJB4_THEEB     ----------------------------------MTTA-TATPVLDYHSDRYKDAYSRIN
tr|V5V382|V5V382_9CHRO     ----------------------------------MTTA-TATPDLDYHSDRYKDAYSRIN
tr|B0C9L0|B0C9L0_ACAM1     ----------------------------------MPQT-QAISEIDFYSDTYKDAYSRID
tr|B8HSZ3|B8HSZ3_CYAP4     ----------------------------------MPQV-QSPSAIDFYSETYQDAYSRID
tr|L8LLH3|L8LLH3_9CHRO     ----------------------------------MQEL-TMPRELDYQSPEYKDAYSRIN
tr|K9Z5G1|K9Z5G1_CYAAP     ----------------------------------MQQ--LVEPTIDFNSDVYKDAYSRIN
tr|I4FNH9|I4FNH9_MICAE     ----------------------------------MPEL-AVPLELDFTSETYKSAYSRIN
tr|I4I7T6|I4I7T6_9CHRO     ----------------------------------MPEL-AVPLELDFTSETYKSAYSRIN
tr|I4HKI6|I4HKI6_MICAE     ----------------------------------MPEL-AVPLELDFTSETYKSAYSRIN
tr|B0JY93|B0JY93_MICAN     ----------------------------------MPEL-AVPLELDFTSETYKSAYSRIN
tr|I4GYG9|I4GYG9_MICAE     ----------------------------------MPEL-AVPLELDFTSETYKSAYSRIN
tr|I4IM50|I4IM50_MICAE     ----------------------------------MPEL-AVSSELDFTSETYKSAYSRIN
tr|I4FD05|I4FD05_MICAE     ----------------------------------MPEL-AVPLELDFTSETYKSAYSRIN
tr|L7E3D3|L7E3D3_MICAE     ----------------------------------MPEL-AVPLELDFTSETYKSAYSRIN
tr|L8NPI8|L8NPI8_MICAE     ----------------------------------MPEL-AVPLELDFTSETYKSAYSRIN
tr|A8YJD6|A8YJD6_MICAE     ----------------------------------MPEL-AVPLELDFTSETYKSAYSRIN
tr|S3JGS6|S3JGS6_MICAE     ----------------------------------MPEL-AVPLELDFTSETYKSAYSRIN
tr|I4GKL8|I4GKL8_MICAE     ----------------------------------MPEL-AVPLELDFTSETYKSAYSRIN
tr|I4HLI6|I4HLI6_MICAE     ----------------------------------MPEL-AVPLELDFTSETYKSAYSRIN
tr|I4FY74|I4FY74_MICAE     ----------------------------------MPEL-AVPLELDFTSETYKSAYSRIN
tr|I4H417|I4H417_MICAE     ----------------------------------MPEL-AVPLELDFTSETYKSAYSRIN
tr|M1LIQ1|M1LIQ1_9SYNC     ----------------------------------MPEL-AVRTEFDYSSEIYKDAYSRIN
tr|H0NYN3|H0NYN3_9SYNC     ----------------------------------MPEL-AVRTEFDYSSEIYKDAYSRIN
tr|H0PB35|H0PB35_9SYNC     ----------------------------------MPEL-AVRTEFDYSSEIYKDAYSRIN
tr|H0PG18|H0PG18_9SYNC     ----------------------------------MPEL-AVRTEFDYSSEIYKDAYSRIN
tr|F7US88|F7US88_SYNYG     ----------------------------------MPEL-AVRTEFDYSSEIYKDAYSRIN
sp|Q55688|ALDEC_SYNY3      ----------------------------------MPEL-AVRTEFDYSSEIYKDAYSRIN
tr|D3EPA5|D3EPA5_ATETH     ----------------------------------MQEL-ALRSELDFNSETYKDAYSRIN
tr|B7JUH1|B7JUH1_CYAP8     ----------------------------------MQEL-VQRSELDFTNPTYKDAYSRIN
tr|C7QUV0|C7QUV0_CYAP0     ----------------------------------MQEL-VQRSELDFTNPTYKDAYSRIN
tr|G5J0P0|G5J0P0_CROWT     ----------------------------------MQEL-AVRSELDFNSETYKDAYSRIN
tr|T2JF13|T2JF13_CROWT     ----------------------------------MQEL-AVRSELDFNSETYKDAYSRIN
tr|Q4C7T3|Q4C7T3_CROWT     ----------------------------------MQEL-AVRSELDFNSETYKDAYSRIN
tr|T2IH71|T2IH71_CROWT     ----------------------------------MQEL-AVRSELDFNSETYKDAYSRIN
tr|A3INE1|A3INE1_9CHRO     ----------------------------------MQEL-ALRSELDFNSETYKDAYSRIN
tr|B1WR71|B1WR71_CYAA5     ----------------------------------MQEL-ALRSELDFNSETYKDAYSRIN
tr|U5DFA3|U5DFA3_9CHRO     ----------------------------------MQEL-AASTALDYDSDVYKDAYSRIN
tr|L8N0G8|L8N0G8_9CYAN     ------------------------------MGQ-----TLEAVAIDYQTENYRDAYSRIN
tr|K9SPY2|K9SPY2_9CYAN     -----------------------------MIGQQ----LDIVTELDYNSDTYRDAYSRIN
tr|K9SZC3|K9SZC3_9SYNE     -----------------------------MIGQIFAAFADENIDIDYRSDTYRDAYSRIN
tr|B4WJ48|B4WJ48_9SYNE     ----------------------------------MQTL-EVSPAMDFQSETYKDAYSRIN
tr|U9WCI1|U9WCI1_9CYAN     ----------------------------------MPQL-EASPTLDFQSEAYKDAYSRIN
tr|W5U411|W5U411_9CYAN     ----------------------------------MPQL-EASLALDFQSEAYKDAYSRIN
tr|K9F0G4|K9F0G4_9CYAN     ----------------------------------MPQL-EASPALDFQSEAYKDAYSRIN
tr|K9ULL9|K9ULL9_9CHRO     ----------------------------------MVQM-AASLELNFTSEIYKDAYSRID
tr|K8GFP8|K8GFP8_9CYAN     ----------------------------------MPQL-EASVAIDFRSEAYRDAYSRIN
tr|D4TEY2|D4TEY2_9NOST     ----------------------------------MQQLVEQIEKTGFQSAKYKDAYSRIN
tr|D4TRY1|D4TRY1_9NOST     ----------------------------------MQQLVEQIEKTDFQSAKYKDAYSRIN
tr|K9V615|K9V615_9CYAN     ----------------------------------MQQL-EAELKIDFNSEIYKDAYSRIN
tr|K9PTP6|K9PTP6_9CYAN     ----------------------------------MQQLTEQ-SEIDFQSEIYKDSYSRIN
tr|K7WTY7|K7WTY7_9NOST     ----------------------------------MQQLVDQIEKIDFQSKIYKDAYSRIN
tr|K9WRL8|K9WRL8_9NOST     ----------------------------------MQQIADQVEKMDFQSETYKDAYSRIN
tr|K9ZJT3|K9ZJT3_ANACC     ----------------------------------MQQLVEQIEKIDFQSEEYKDAYSRIN
tr|D7E2W1|D7E2W1_NOSA0     ----------------------------------MQQLVEEIEKIDFQSEKYKDAYSRIN
tr|Q112R8|Q112R8_TRIEI     ------MPKLEIIPTMD--S-------QSETKLEKVKSQSEGDQINFETETYKDAYSRIN
sp|Q54764|ALDEC_SYNE7      ----------------------------------MPQL-EASLELDFQSESYKDAYSRIN
tr|Q8KPT4|Q8KPT4_SYNE7     ----MRTPWDPPNPTFSLSS-------VSGDRRLMPQL-EASLELDFQSESYKDAYSRIN
tr|Q5N627|Q5N627_SYNP6     ----MRTPWDPPNPTFSLSS-------VSGDRRLMPQL-EASLELDFQSESYKDAYSRIN
tr|K9RFH5|K9RFH5_9CYAN     ----------------------------------MQEL-ISPSELDYRSETYKSAFSRIN
tr|K9TXF6|K9TXF6_9CYAN     ----------------------------------MQQV-AATSEIDFHSETYKDAYSRIN
tr|K9X9I4|K9X9I4_9CHRO     ----------------------------------MQQLAATAAELDFHSETYKDAYSRIN
tr|A0Z9X5|A0Z9X5_NODSP     ----------------------------------MQQL-AAELKIDFQSEKYKDAYSRIN
tr|Q3MA38|Q3MA38_ANAVT     ----------------------------------MQQV-AADLEIDFKSEKYKDAYSRIN
```

```
                                                          1.......10........20......
                                                                      : .  *:.:: *::
tr|Q8YLL5|Q8YLL5_NOSS1       ----------------------------------MQQV-AADLEIDFKSEKYKDAYSRIN
tr|K9QXA2|K9QXA2_NOSS7       ----------------------------------MQQI-AGDLEIDFKSEKYKDAYSRIN
tr|M1X0T0|M1X0T0_9NOST       --------------------------------MTMQQL-ATQSEIDFSSEIYKDAYSRIN
tr|M1WVU9|M1WVU9_9NOST       ----------------------------------MQQL-ATQSEIDFSSEIYKDAYSRIN
tr|K9S7C1|K9S7C1_9CYAN       ----------------------------------MPQL-EASLELDFHSETYKDAYSRIN
tr|K9W4T2|K9W4T2_9CYAN       ----------------------------------MAQ---IAASIDFQSETYKDAYSRIN
tr|G6FV22|G6FV22_9CYAN       ----------------------------------MQQL-ADQPEIDFHSETYKDAYSRIN
tr|W5U379|W5U379_9CYAN       -----------------MYH-------IFRSGRTMQQL-ANQPDIDFKSETYKDAYSRVN
tr|W5U417|W5U417_9CYAN       -----------------MYH-------IFRSGRTMQQL-ANQPDIDFKSETYKDAYSRVN
tr|K9QA48|K9QA48_9NOSO       ----------------------------------MQQL-AAELEIDFQSETYKDAYSRIN
tr|G9HTG5|G9HTG5_9NOSO       ----------------------------------MQPL-AAELEIDFQSETYKDAYSRIN
tr|H2DDT7|H2DDT7_NOSS6       ----------------------------------MQPL-AAELEIDFQSETYKDAYSRIN
tr|K9YV86|K9YV86_DACSA       ----------------------------------MQD-VAVTPTLDYTSETYKDAYSRIN
tr|K9Y9U4|K9Y9U4_HALP7       ----------------------------------MQD-VAVTPTLDYNSETYKDAYSRIN
tr|W5U332|W5U332_PLAAG       ----------------------------------MPQVEA--ITIDFQSETYKDAYSRIN
tr|F5UGX9|F5UGX9_9CYAN       ----------------------------------MPQLEA-SPTIDFQTETYKDAYSRIN
tr|K9VE50|K9VE50_9CYAN       ----------------------------------MPQLEA-SPTIDFQTETYKDAYSRIN
tr|D8FVZ2|D8FVZ2_9CYAN       ----------------------------------MQQLEA-SPAIDFETATYKDAYSRIN
tr|B4VSW8|B4VSW8_9CYAN       -------------------M-------QTGENLLMQQLTV-SQELDFNSETYKDAYSRIN
tr|K9WII2|K9WII2_9CYAN       ----------------------------------MQQLAV-SPEFDFTSESYKDAYSRIN
tr|K9TGS5|K9TGS5_9CYAN       ----------------------------------MPQLEATSATLDFQSPTYKDAYSRIN
tr|B5W0S5|B5W0S5_ARTMA       ----------------------------------MPQLETI-TELDFQNETYKDAYSRIN
tr|K1WZT6|K1WZT6_ARTPT       ----------------------------------MPQLETI-TELDFQNETYKDAYSRIN
tr|W6SNU7|W6SNU7_9CYAN       ----------------------------------MPQLETI-TELDFQNETYKDAYSRIN
tr|H1WAD2|H1WAD2_9CYAN       ----------------------------------MPQLETI-TELDFQNETYKDAYSRIN
tr|K6DQI0|K6DQI0_ARTPT       ----------------------------------MPQLETI-AELDFQNETYKDAYSRIN
tr|D5A1Z8|D5A1Z8_ARTPN       ----------------------------------MPQLETI-AELDFQNETYKDAYSRIN
tr|U7QM55|U7QM55_9CYAN       ----------------------------------MPQLEAI-AEIDFNTNTYKDAYSRIN
tr|A0YKK4|A0YKK4_LYNSP       ----------------------------------MPQLEAI-AEIDFNTNTYKDAYSRIN
tr|A3Z6M0|A3Z6M0_9SYNE       -----------------------------------------MTQLDFASAAYREAYSRIN
tr|B5ILL6|B5ILL6_9CHRO       ------------MASVA----HPAVAVQPATKPADTAAERGDGLPDFSSDTYKDAYSRIN
tr|A3YYU8|A3YYU8_9SYNE       ------------MPSLETTIAASETASASASMAVGGSVEQDLGLPDFSSSTYKDAYSRIN
tr|A2C7S0|A2C7S0_PROM3       ------------MPTLEMPE----------AAVLDSTVGSSEALPDFTSDAYKDAYSRIN
sp|Q7V6D4|ALDEC_PROMM        ------------MPTLEMPV----------AAVLDSTVGSSEALPDFTSDRYKDAYSRIN
tr|A5GUD0|A5GUD0_SYNR3       -----------------MAS-------SLLDPAVDGTPVLDVELPDFTTEAYKSAYSRIN
tr|K9P3X5|K9P3X5_CYAGP       ------------MPTVATTT-------SVDSSLDPVGPIEGSDLPDFSTAAYKDAYSRIN
tr|Q0I8M9|Q0I8M9_SYNS3       ------------MPTLDSTL----------VAVLDDQQGL-AELPDFTTDAYKDAYSRIN
tr|G4FIH8|G4FIH8_9SYNE       ------------MSTLDSTA----------VAVLDDQQGL-AELPDFTTDAYKDAYSRIN
tr|Q05RJ5|Q05RJ5_9SYNE       ------------MPTLNSPE----------VAAISDQDGSASQLPDFSSAAYKDAYSRIN
tr|A5GJG5|A5GJG5_SYNPW       ------------MPTPVTSE----------VAVLDGQAGSAQALPDFSSEAYKDAYSRIN
tr|A4CS34|A4CS34_SYNPV       ------------MPTPVTSE----------VAVLDEQAGSASLLPDFSSEAYKDAYSRIN
tr|A3Z5H6|A3Z5H6_9SYNE       ------------MPTLETSE----------VAVLEDSMASGSRLPDFTSEAYKDAYSRIN
tr|Q060S1|Q060S1_9SYNE       --------------------------------MLEG----QDALPDFTTAEYKDAYSRIN
tr|Q3AV61|Q3AV61_SYNS9       ------------MPTLNAPE----------VSVLEG----QDALPDFTTAEYKDAYSRIN
tr|Q0QKQ4|Q0QKQ4_9SYNE       ------------MTTLNAPE----------ASVMEG----QDALPDFTTEAYKDAYSRIN
tr|Q0QM11|Q0QM11_9SYNE       ------------MTTLNAPD----------AAVVEG----LDALPDFTTEAYKDAYSRIN
tr|Q3ALN2|Q3ALN2_SYNSC       ------------MTTLNAPE----------APVLEG----QDALPDFTTAAYKDAYSRIN
tr|W0GVR9|W0GVR9_9SYNE       ------------MTTLNAPE----------APVLEG----QDALPDFTTEAYKDAYSRIN
tr|Q0QKF3|Q0QKF3_9SYNE       ------------MTTLNAPE----------AAVVEG----LDALPDFTTEAYKDAYSRIN
tr|Q7U5G9|Q7U5G9_SYNPX       ------------MTTLNAPE----------AAVVEG----LDALPDFTTEAYKDAYSRIN
tr|Q0QKK1|Q0QKK1_9SYNE       ------------MTTLNAPE----------AAVVEG----LDALPDFTTEAYKDAYSRIN
tr|A2C0Z0|A2C0Z0_PROM1       ------------MQAFASNN---------LT--VEKEELSSDSLPDFTSESYKDAYSRIN
tr|Q46GP5|Q46GP5_PROMT       ------------MQAFASNN---------LT--VEKEELSSNSLPDFTSESYKDAYSRIN
tr|A8G3P7|A8G3P7_PROM2       -----MNKSLTDMQTLESKK---------DIQLEGSTDNDSANLPDFTTDAYKDAYSRIN
tr|A2BVJ4|A2BVJ4_PROM5       ------------MQTLESNK---------NIQIGSSPESDSANLPDFTTDAYKDAYSRIN
tr|Q7V2E8|Q7V2E8_PROMP       ------------MQTLESNK---------KT-N----LENSIDLPDFTTDSYKDAYSRIN
tr|Q31C02|Q31C02_PROM9       MH---NELKITDMQTLETNT---------KT-TEESIDTNSLNLPDFTTDSYKDAYSRIN
tr|A2BQ13|A2BQ13_PROMS       MH---NELKITDMQTLESNK---------KT-IEESINPISLDLPDFTTDSYKDAYSRIN
tr|A3PBQ6|A3PBQ6_PROM0       MH---NELKITDMQTLESNK---------KT-IEESTNSISLDLPDFTTDSYKDAYSRIN
tr|A9BEF6|A9BEF6_PROM4       ------------MPTLESSE---------VA-VISDLEGRDGSLPDFTTEQYKDAYSRIN
tr|Q7VD51|Q7VD51_PROMA       ------------MQTLTN---------------QVASADELDNLPDFSSSQYKDAYSRIN
tr|B8HLW2|B8HLW2_CYAP4       -----------------------------------MSDCATNPALDYYSETYRNAYRRVN
tr|U9VZT6|U9VZT6_9CYAN       -----------------------------------MSHSVADQTLDFYSDTYRDAFTRIN
tr|K9EQR0|K9EQR0_9CYAN       -----------------------------------MPYSAVDQTLDFHSDTYRDAFTRIN
```

```
                            ..30........40........50........60........70........80......
                            .:*: **  *  .:  :   :      :  *. **  *  .* .*..** :  *   .
sp|B2J1M1|ALDEC_NOSP7       AIVIEGEQEAHENYITLAQLLPESHDELIRLSKMESRHKKGFEACGRNLAVTPDLQFAKE
tr|U5QDK0|U5QDK0_9CYAN      AIVLEGEREACANYLALAELLPDHADALKKLSAMENRHFKGFQSCARNLEVTPDDEFARR
tr|Q7NGM3|Q7NGM3_GLOVI      AIVLEGEREAHANYLTLAEMLPDHAEALKKLAAMENRHFKGFQSCARNLEVTPDDPFARA
tr|Q2JJ13|Q2JJ13_SYNJB      GIVIEGEQEAHDNYLKLAEMLPEGAEELHKLAKMELRHMKGFQSCGKNLQVEPDREFART
tr|Q2JX62|Q2JX62_SYNJA      GIVIEGEQEAHDNYLKLAEMLPEAAEELRKLAKMELRHMKGFQACGKNLQVEPDVEFARA
tr|K9RTZ6|K9RTZ6_SYNP3      GIVIEGEKEAHENYISLTKLIPDYEEDLLRLSKMEARHKKGFEACGRNLAVSPDLDFAEK
tr|Q8DJB4|Q8DJB4_THEEB      AIVIEGEQEAHDNYIDLAKLLPQHQEELTRLAKMEARHKKGFEACGRNLSVTPDMEFAKA
tr|V5V382|V5V382_9CHRO      AIVIEGEQKACDNYIDLAKLLPQHQEELTRLAKMEARHKKGFEACGRNLNVTPDMGFAKA
tr|B0C9L0|B0C9L0_ACAM1      GIVIEGEQEAHENYIRLGEMLPEHQDDFIRLSKMEARHKKGFEACGRNLKVTCDLDFARR
tr|B8HSZ3|B8HSZ3_CYAP4      AIVIEGEQEAHDNYLKLTELLPDCQEDLVRLAKMEARHKKGFEACGRNLKVTPDMEFAQQ
tr|L8LLH3|L8LLH3_9CHRO      AIVIEGEHEAYQNYITLGDMLPDVQAELVRLAKMESRHAKGFQSCGKNLNVTPDMEYAKK
tr|K9Z5G1|K9Z5G1_CYAAP      AIVIEGEQEAHDNYMDMIPMLPNHEKELASLAKMEFRHKKGFQSCGRNLSVTPDMDFAQK
tr|I4FNH9|I4FNH9_MICAE      AIVIEGEYEANSNYIQLADILTDNKEELHRLAKMENRHMKGFQACGRNLQITPDMDYARA
tr|I4I7T6|I4I7T6_9CHRO      AIVIEGEYEANSNYIQLADILADNKEELHRLAKMENRHMKGFQACGQNLQITPDMDYARE
tr|I4HKI6|I4HKI6_MICAE      AIVIEGEYEANSNYIQLADILTDNKEELHRLAKMENRHMKGFQACGQNLKITPDMDYARE
tr|B0JY93|B0JY93_MICAN      AIVIEGEYEANSNYIQLADILTDNKEELHRLAKMENRHMKGFQACGQNLKITPDMDYARE
tr|I4GYG9|I4GYG9_MICAE      AIVIEGEYEANSNYIQLADILTDNKEELHRLAKMENRHMKGFQACGQNLQITPDMDYARE
tr|I4IM50|I4IM50_MICAE      AIVIEGEYEANSNYIQLADILTDNKEELHRLAKMENRHMKGFQACGQNLQITPDMDYARE
tr|I4FD05|I4FD05_MICAE      AIVIEGEYEANSNYIQLADILTDNKEELHRLAKMENRHMKGFQACGQNLQITPDMDYARE
tr|L7E3D3|L7E3D3_MICAE      AIVIEGEYEANSNYIQLADILTDNKEELHRLAKMENRHMKGFQACGQNLQITPDMDYARE
tr|L8NPI8|L8NPI8_MICAE      AIVIEGEYEANSNYIQLADILTDNKEELHRLAKMENRHMKGFQACGQNLQITPDMEYAKE
tr|A8YJD6|A8YJD6_MICAE      AIVIEGEYEANSNYIQLADILTDNKEELHRLAKMENRHMKGFQACGQNLQITPDMEYAKE
tr|S3JGS6|S3JGS6_MICAE      AIVIEGEYEANSNYIQLADILTDNKEELHRLAKMENRHMKGFQACGQNLKITPDMEYAKE
tr|I4GKL8|I4GKL8_MICAE      AIVIEGEYEANSNYIQLADILTDNKEELHRLAKMENRHMKGFQACGQNLKIIPDMEYAKE
tr|I4HLI6|I4HLI6_MICAE      AIVIEGEYEANSNYIQLADILTDNKEELHRLAKMENRHMKGFQACGQNLKITPDMEYAKE
tr|I4FY74|I4FY74_MICAE      AIVIEGEYEANSNYIQLADILTDNKEELHRLAKMENRHMKGFQACGRNLQITPDMDYAKE
tr|I4H417|I4H417_MICAE      AIVIEGEYEANSNYIQLADILTDKKEELHRLAKMENRHMKGFQACGRNLQITPDMDYAKE
tr|M1LIQ1|M1LIQ1_9SYNC      AIVIEGEQEAYSNYLQMAELLPEDKEELTRLAKMENRHKKGFQACGNNLQVNPDMPYAQE
tr|H0NYN3|H0NYN3_9SYNC      AIVIEGEQEAYSNYLQMAELLPEDKEELTRLAKMENRHKKGFQACGNNLQVNPDMPYAQE
tr|H0PB35|H0PB35_9SYNC      AIVIEGEQEAYSNYLQMAELLPEDKEELTRLAKMENRHKKGFQACGNNLQVNPDMPYAQE
tr|H0PG18|H0PG18_9SYNC      AIVIEGEQEAYSNYLQMAELLPEDKEELTRLAKMENRHKKGFQACGNNLQVNPDMPYAQE
tr|F7US88|F7US88_SYNYG      AIVIEGEQEAYSNYLQMAELLPEDKEELTRLAKMENRHKKGFQACGNNLQVNPDMPYAQE
sp|Q55688|ALDEC_SYNY3       AIVIEGEQEAYSNYLQMAELLPEDKEELTRLAKMENRHKKGFQACGNNLQVNPDMPYAQE
tr|D3EPA5|D3EPA5_ATETH      AIVIEGEQEAYQNYLDMVHMLPKNKDELVRLSKMENRHKTGFQACGKNLNVIPDMQYAKE
tr|B7JUH1|B7JUH1_CYAP8      AIVIEGEQEAHQNYIDMAQLLPEHQEELIRLSKMENRHKKGFEACGNNLSVTPDMQYAQE
tr|C7QUV0|C7QUV0_CYAP0      AIVIEGEQEAHQNYIDMAQLLPEHQEELIRLSKMENRHKKGFEACGNNLSVTPDMQYAQE
tr|G5J0P0|G5J0P0_CROWT      AIVIEGEQEAYENYIDMGELLPGDKDELIRLSKMENRHKKGFQACGKNLKVTPDMDYAER
tr|T2JF13|T2JF13_CROWT      AIVIEGEQEAYENYIDMGELLPGDKDELIRLSKMENRHKKGFQACGKNLKVTPDMDYAER
tr|Q4C7T3|Q4C7T3_CROWT      AIVIEGEQEAYENYIDMGELLPGDKDELIRLSKMENRHKKGFQACGKNLKVTPDMDYAER
tr|T2IH71|T2IH71_CROWT      AIVIEGEQEAYENYIDMGELLPGDKDELIRLSKMENRHKKGFQACGKNLKVTPDMDYAER
tr|A3INE1|A3INE1_9CHRO      AIVIEGEQEAHQNYIDMAQLLPEDEAELIRLSKMENRHKKGFQACGKNLDVTPDMDYAQQ
tr|B1WR71|B1WR71_CYAA5      AIVIEGEQEAYQNYLDMAQLLPEDEAELIRLSKMENRHKKGFQACGKNLNVTPDMDYAQQ
tr|U5DFA3|U5DFA3_9CHRO      AIVIEGEMEAKGNYTQLATLLPDSAEDLTQLAKMEGRHMKGFQACGKNLSVTPDMDFARD
tr|L8N0G8|L8N0G8_9CYAN      AIVIEGEAEAHSNYLQLAELMPEFNEELHRLAKMEDRHKKGFTACGKNLNVTPDMDFAEK
tr|K9SPY2|K9SPY2_9CYAN      AIVIEGEQEAYSNYLRLGEMLPDLKDDLAKLAKMEMRHKKGFTACGKNLSVTPDMQFAQE
tr|K9SZC3|K9SZC3_9SYNE      AIVIEGEQEAHDNYLQLGELLPELKTELAGLAKMENRHKKGFEACGRNLSVIPDMQFAKE
tr|B4WJ48|B4WJ48_9SYNE      AIVIEGELEANNNYKQLSEHLGDFKDDLLKLARMENRHMKGFQACGKNLSVNPDMPFAKE
tr|U9WCI1|U9WCI1_9CYAN      AIVIEGELEANDNYKKLAENLNEHKDELIKLARMENRHMKGFQACGKNLNVTPDMEFAKI
tr|W5U411|W5U411_9CYAN      AIVIEGEWEANDNYQTLAEHLSDHKDELIKLARMEKRHMKGFQACGKNLNVTPDMKFAKE
tr|K9F0G4|K9F0G4_9CYAN      AIVIEGEWEANDNYQKLAEHLGEHKDELIKLARMEKRHMKGFQACGKNLNVTPDMKFAKE
tr|K9ULL9|K9ULL9_9CHRO      AIVIEGEQEAHENYITLSGLLPDFQDDLVKLSKMELRHKKGFEACARNLAVTPDLGFAKE
tr|K8GFP8|K8GFP8_9CYAN      AIVIEGEQEANENYVKLAELLPDSKEQLLGLAKMESRHKKGFEACGRNLEVTPDMAFARE
tr|D4TEY2|D4TEY2_9NOST      AIVIEGEQEAHENYLTLGELLPPNKEELIKLSKMESRHKKGFESCGRNLSVNPDMEFARQ
tr|D4TRY1|D4TRY1_9NOST      AIVIEGEQEAHENYLTLGELLPPNKEELSKLSKMESRHKKGFESCGRNLSVNPDMEFARQ
tr|K9V615|K9V615_9CYAN      AIVIEGEQEAHENYISLGELLPDSKDELVRLSKMESRHKKGFEACGRNLKVTPDMGFARE
tr|K9PTP6|K9PTP6_9CYAN      AIVIEGEQEAYDNYIKLAELLPENQAELIRLSKMESRHKKGFEACGRNLQVVPDLEFAKA
tr|K7WTY7|K7WTY7_9NOST      AIVIEGEQEAHENYMTLAKLLPAQEEELIRLSKMESRHKKGFEACGRNLKVTPDLVFAKK
tr|K9WRL8|K9WRL8_9NOST      AIVIEGEQEAHDNYIKLAELLPTKQDELIRLSKMESRHKKGFEACGRNLKVTPDMEFAQN
tr|K9ZJT3|K9ZJT3_ANACC      AIVIEGEQEAHDNYIQLAELLPESKDNLIRLSKMESRHKKGFEACGRNLQVTPDMKFAKE
tr|D7E2W1|D7E2W1_NOSA0      AIVIEGEQEAHENYITLAKLLPESKEELMRLSKMESRHKKGFEACGRNLQVTPDMQFAKE
tr|Q112R8|Q112R8_TRIEI      AIVIEGEQEAYKNYIKLAEMLPDEKDELIKLSKMENRHKKGFEACGRNLHVTPDMEFAKK
sp|Q54764|ALDEC_SYNE7       AIVIEGEQEAFDNYNRLAEMLPDQRDELHKLAKMEQRHMKGFMACGKNLSVTPDMGFAQK
tr|Q8KPT4|Q8KPT4_SYNE7      AIVIEGEQEAFDNYNRLAEMLPDQRDELHKLAKMEQRHMKGFMACGKNLSVTPDMGFAQK
tr|Q5N627|Q5N627_SYNP6      AIVIEGEQEAFDNYNRLAEMLPDQRDELHKLAKMEQRHMKGFMACGKNLSVTPDMGFAQK
tr|K9RFH5|K9RFH5_9CYAN      AIVVEGEQEAYENYLSLGQLLPDKKDDLVRLSKMEMRHKKGFQACAKNLEVTADMEFAKE
tr|K9TXF6|K9TXF6_9CYAN      AIVIEGEQEAYENYIRLAQMLPNLKDELIRLSKMENRHKKGFEACGRNLQVTPDMQFARE
tr|K9X9I4|K9X9I4_9CHRO      AIVIEGEKEAHENYIRLAELLPEHKEELIRLSKMENRHKKGFEACGRNLQVTPDMQFAQE
tr|A0Z9X5|A0Z9X5_NODSP      AIVIEGEQEAHDNYITLGEMLPELKDELIRLSKMESRHKKGFEACGRNLSVKPDMPFAQK
tr|Q3MA38|Q3MA38_ANAVT      AIVIEGEQEAYENYIQLSQLLPDDKEDLIRLSKMESRHKKGFEACGRNLQVSPDIEFAKE
```

```
                                    ..30........40........50........60........70........80......
                                    .:*: **  *  .:  :   :      :  *. **  *  .* .*..** :  *   .
tr|Q8YLL5|Q8YLL5_NOSS1              AIVIEGEQEAYENYIQLSQLLPDDKEDLIRLSKMESRHKKGFEACGRNLQVSPDMEFAKE
tr|K9QXA2|K9QXA2_NOSS7              AIVIEGEQEAYDNYIQLAEMLPEHKDELIRLSKMENRHKKGFEACGRNLEVSPDMQFAKE
tr|M1X0T0|M1X0T0_9NOST              AIVIEGEQEAHENYITLAEMLPEENEQLIRLSQMESRHKKGFEACGRNLQVAPDMKFAKK
tr|M1WVU9|M1WVU9_9NOST              AIVIEGEQEAHENYITLAEMLPEENEQLIRLSQMESRHKKGFEACGRNLQVAPDIKFAKN
tr|K9S7C1|K9S7C1_9CYAN              AIVIEGEQEAYENYLKLAELLPDNQDELVRLSKMESRHKKGFQACGRNLEVTPDMDFAKA
tr|K9W4T2|K9W4T2_9CYAN              AIVIEGEQEAYENYTKLAELLPESKDELIRLSKMEMRHKKGFEACGRNLQVTADLEFARN
tr|G6FV22|G6FV22_9CYAN              AIVIEGEQEAHENYLKLAELLSEHKDELIRLAKMESRHKKGFEACGRNLQVTPDIEFAKQ
tr|W5U379|W5U379_9CYAN              AIVIEGEQEAHENYLKLAELLSEHKDDLIRLSKMESRHKKGFEACGRNLQVTPDLEFAKQ
tr|W5U417|W5U417_9CYAN              AIVIEGEQEAHENYLKLAELLSEHKDDLIRLSKMESRHKKGFEACGRNLQVTPDLEFAKQ
tr|K9QA48|K9QA48_9NOSO              AIVIEGEQEAYENYIKLAELLPANHDQLIGLSKMESRHKKGFEACGRNLKVTPDLQFAKE
tr|G9HTG5|G9HTG5_9NOSO              AIVIEGEQEAYENYIKLAELLPANHDQLIGLSKMESRHKKGFEACGRNLKVTPDLQFAKE
tr|H2DDT7|H2DDT7_NOSS6              AIVIEGEQEAYENYIKLAELLPANHDQLIGLSKMESRHRKGFEACGRNLKVTPDLQFAKE
tr|K9YV86|K9YV86_DACSA              AIVIEGEQEAKDNYLHLIQLLPDSKDELTSLSKMEARHKKGFQACGKNLSVTPDMEFAQE
tr|K9Y9U4|K9Y9U4_HALP7              AIVIEGEQEAKDNYLHLIELLPDSKEELTRLSKMEARHKKGFQACGKNLNVTPDMEFAQE
tr|W5U332|W5U332_PLAAG              AIVIEGEQEAYDNYIKLGAMLPEQQENLAGLAKMEMSHKRGFQSCGRNLTVTPDMEFAQD
tr|F5UGX9|F5UGX9_9CYAN              AIVIEGEQEAHDNYLTLAELLADKKAELIGLSKMENRHMKGFQACGRNLKVTPDMAFAKQ
tr|K9VE50|K9VE50_9CYAN              AIVIEGEQEAHDNYLTLAELLADKKAELVGLSKMENRHMKGFQACGRNLKVTPDMAFAKE
tr|D8FVZ2|D8FVZ2_9CYAN              AIVIEGEQEAYDNYIRLGEMLPDQKDVLIALSKMENRHMKGFQACGRNLKVTPDMEFAKD
tr|B4VSW8|B4VSW8_9CYAN              AIVIEGEQEAHQNYIQLAELLPDQKDELTSLAKMENRHKKGFQACGRNLSVTADMEFAKE
tr|K9WII2|K9WII2_9CYAN              AIVIEGEQEAHDNYITLAQLLPDHKDELQRLSKMESRHMKGFQACGRNLSVTPDMEFAKQ
tr|K9TGS5|K9TGS5_9CYAN              AIVIEGEQEANDNYIKLAEMLPDCKDELLGLAKMELRHKKGFQACGRNLAVEPDMEFARE
tr|B5W0S5|B5W0S5_ARTMA              AIVIEGEQEASDNYIKLGEMLPEEREELIRLSKMEKRHKKGFQACGRNLEVTPDMDFGRE
tr|K1WZT6|K1WZT6_ARTPT              AIVIEGEQEASDNYIKLGEMLPEEREELIRLSKMEKRHKKGFQACGRNLEVTPDMDFGRE
tr|W6SNU7|W6SNU7_9CYAN              AIVIEGEQEASDNYIKLGEMLPEEREELIRLSKMEKRHKKGFQACGRNLEVTPDMDFGRE
tr|H1WAD2|H1WAD2_9CYAN              AIVIEGEQEASDNYIKLGEMLPEEREELIRLSKMEKRHKKGFQACGRNLEVTPDMDFGRE
tr|K6DQI0|K6DQI0_ARTPT              AIVIEGEQEASDNYIKLGEMLPEEREELIRLSKMEKRHMKGFQACGRNLEVSPDMDFGRE
tr|D5A1Z8|D5A1Z8_ARTPN              AIVIEGEQEAYDNYIKLGEMLPEEREELIRLSKMEKRHMKGFQACGRNLEVSPDMDFGRE
tr|U7QM55|U7QM55_9CYAN              AIVIEGEQEAHDNYIKLGEMLPHEKDELVRLSKMEKRHMKGFQACGRNLEVTPDMGYARQ
tr|A0YKK4|A0YKK4_LYNSP              AIVIEGEQVAHDNYIKLGEMLPDQKDELVRLSKMEKRHMKGFQACGRNLEVTADMDYAHQ
tr|A3Z6M0|A3Z6M0_9SYNE              GVVIVGEGLANRHFQMLARRIPADRDELQRLGRMEGDHASAFVGCGRNLGVVADLPLARR
tr|B5ILL6|B5ILL6_9CHRO              AIVIEGEQEAHDNYIALGTLIPDQADELAKLARMELKHMKGFTACANNLGVTADMPFAKE
tr|A3YYU8|A3YYU8_9SYNE              AIVIEGEQEAHDNYISLGHLIPDQAEELERLARMELKHKKGFTACAKNLSVIADMDFAKE
tr|A2C7S0|A2C7S0_PROM3              AIVIEGEQEAHDNYIAIGTLLPDHVEELKRLAKMEMRHKKGFTACGKNLGVTADMDFARE
sp|Q7V6D4|ALDEC_PROMM               AIVIEGEQEAHDNYIAIGTLLPDHVEELKRLAKMEMRHKKGFTACGKNLGVEADMDFARE
tr|A5GUD0|A5GUD0_SYNR3              AIVIEGEQEAHDNYISLGTLIPDQADELAQLARMEMKHMKGFQACGKNLSVEPDMVFAKE
tr|K9P3X5|K9P3X5_CYAGP              AIVIEGEQEAHDNYMSLGTLLPDQAEELARLARMELKHMKGFTACGNNLGVTADMPFAQE
tr|Q0I8M9|Q0I8M9_SYNS3              AIVIEGEKEAHDNYLSLGTLIPEQAEELAKLAKMEMKHMKGFTACAKNLDVVADMPFAQE
tr|G4FIH8|G4FIH8_9SYNE              AIVIEGEQEAHDNYISLGTLIPDQAEELAKLAKMEMKHMKGFTACAKNLNVVADMPFAQE
tr|Q05RJ5|Q05RJ5_9SYNE              AIVIEGEQEAHDNYISLGTLIPDQADELKGLARMEMKHMKGFTACGNNLGVTADMDFART
tr|A5GJG5|A5GJG5_SYNPW              AIVIEGEQEAHDNYISLGTLIPEQADELARLARMEMKHMKGFMSCGRNLGVEADMPFAKE
tr|A4CS34|A4CS34_SYNPV              AIVIEGEQEAHDNYISLGTLIPDQADELARLARMEMKHMKGFTSCGRNLGVDADMPFAKT
tr|A3Z5H6|A3Z5H6_9SYNE              AIVIEGEQEAHDNYIALGTLIPEQKDELARLARMEMKHMKGFTSCGRNLGVEADLPFAKE
tr|Q060S1|Q060S1_9SYNE              AIVIEGEQEAHDNYISLGTLIPEQAEELSRLARMEMKHMKGFTACARNLGVEADMPFAKE
tr|Q3AV61|Q3AV61_SYNS9              AIVIEGEQEAHDNYISLGTLIPEQADELSRLARMEMKHMKGFTACARNLGVEADMPFAKD
tr|Q0QKQ4|Q0QKQ4_9SYNE              AIVIEGEQEAHDNYISLGTLIPDQAEELARLARMEMKHMKGFTSCGRNLGVQADMAFARK
tr|Q0QM11|Q0QM11_9SYNE              AIVIEGEQEAHDNYIALGTLIPDQKDELARLARMEMKHMKGFTSCGRNLGVKADMVFAKT
tr|Q3ALN2|Q3ALN2_SYNSC              AIVIEGEQEAHDNYISLGTLIPEQAEELKRLARMEMKHMKGFTSCGRNLGVEADLPFAKK
tr|W0GVR9|W0GVR9_9SYNE              AIVIEGEQEAHDNYISLGTLIPDQAEELKRLARMEMKHMKGFTSCGRNLGVEADLPFAKK
tr|Q0QKF3|Q0QKF3_9SYNE              AIVIEGEQEAHDNYISLGTLIPDQKDELAKLARMEMKHMKGFTSCGRNLGVEADLAFAKK
tr|Q7U5G9|Q7U5G9_SYNPX              AIVIEGEQEAHDNYISLGSLIPDQKDELAKLARMEMKHMKGFTSCGRNLGVEADMVFAKK
tr|Q0QKK1|Q0QKK1_9SYNE              AIVIEGEQEAHDNYISLGSLIPDQKDELAKLARMEMKHMKGFTSCGRNLGVEADMVFAKT
tr|A2C0Z0|A2C0Z0_PROM1              AVVIEGEQEAYSNFLDLAKLIPEHADELVRLGKMEKKHMNGFCACGRNLAVKPDMPFAKT
tr|Q46GP5|Q46GP5_PROMT              AVVIEGEQEAYSNFLDLAKLIPEHADELVRLGKMEKKHMNGFCACGRNLAVKPDMPFAKT
tr|A8G3P7|A8G3P7_PROM2              AIVIEGEQEAYDNYISIATLLPNDSEELTKLAKMELKHKRGFTACGKNLGVEADMPFAKE
tr|A2BVJ4|A2BVJ4_PROM5              AIVIEGEQEAYDNYISIATLLPNDSEELTKLAKMELKHKRGFTACGKNLGVEADMSFAKE
tr|Q7V2E8|Q7V2E8_PROMP              AIVIEGEQEAHDNYISLATLIPNELEELTKLAKMELKHKRGFTACGRNLGVQADMIFAKE
tr|Q31C02|Q31C02_PROM9              AIVIEGEQEAHDNYISIATLIPNELEELTKLARMELKHKKGFTACGRNLGVDADMVFAKK
tr|A2BQ13|A2BQ13_PROMS              AIVIEGEQEAHDNYISIATLIPNEVEELTKLARMEMKHKKGFTACGRNLGVVADMDFAKK
tr|A3PBQ6|A3PBQ6_PROM0              AIVIEGEQEAHDNYISIATLIPNELEELTKLARMEMKHKKGFTACGRNLDVVADMEFAKK
tr|A9BEF6|A9BEF6_PROM4              AIVIEGEKEAHDNYVAIGTVIPEKADELKKLAIMELRHMKGFTACGKNLGVVADMEFAQR
tr|Q7VD51|Q7VD51_PROMA              AIVIEGEKEAHDNYMSIGTLIPDKADELKKLAVMELKHMRGFTACGKNLGVKADIPFAEK
tr|B8HLW2|B8HLW2_CYAP4              GIVIEGEKQAYDNFIRLAELLPEYQAELTRLAKMEARHQKSFVACGQNLKVSPDLDFAAQ
tr|U9VZT6|U9VZT6_9CYAN              GIVIEGEQAAYDNFLCLADLLPEQSEELTRLGKMEGRHRKSFEACGRNLNVIPDLAFANQ
tr|K9EQR0|K9EQR0_9CYAN              GIVIEGEQAAYDNFLCLADLLPEQAEELVRLGKMEGRHRKSFEACGRNLDVIPDLTFANQ
```

```
                                 ..90.......100  .......110.......120.......130.......140....
                                  *  *   *             *:::* :::* **::**. ::  :* ::: :*  *: :
sp|B2J1M1|ALDEC_NOSP7            FFSGLHQNFQTAAA--EGKVVTCLLIQSLIIECFAIAAYNIYIPVADDFARKITEGVVKE
tr|U5QDK0|U5QDK0_9CYAN           YFAGLDANFQRAAA--SGDIAACMVIQALIIECFAIAAYNIYIPVADPFARRVTEGVVKD
tr|Q7NGM3|Q7NGM3_GLOVI           YFEQLDGNFQQAAA--EGDLTTCMVIQALIIECFAIAAYNVYIPVADAFARKVTEGVVKD
tr|Q2JJ13|Q2JJ13_SYNJB           FFAPLRNNFQKAAA--AGDLVTCLVIQSLIIECFAIAAYNIYIPVADEFARKITEGVVKD
tr|Q2JX62|Q2JX62_SYNJA           FFAPLRDNFQSAAA--AGDLVSCFVIQSLIIECFAIAAYNIYIPVADDFARKITEGVVKD
tr|K9RTZ6|K9RTZ6_SYNP3           FFADLHNNFQVAAA--EGKIATCLVIQALIIECFAIAAYNIYIPMADDFARKITEGVVKD
tr|Q8DJB4|Q8DJB4_THEEB           FFEKLRANFQRALA--EGKTATCLLIQALIIESFAIAAYNIYIPMADPFARKITESVVKD
tr|V5V382|V5V382_9CHRO           FFEKLHGNFQAALA--EGKIATCLLIQALIIECFAIAAYNIYIPMADPFARKITEGVVKD
tr|B0C9L0|B0C9L0_ACAM1           FFSDLHKNFQDAAA--EDKVPTCLVIQSLIIECFAIAAYNIYIPVADDFARKITESVVKD
tr|B8HSZ3|B8HSZ3_CYAP4           FFADLHNNFQKAAA--ANKIATCLVIQALIIECFAIAAYNIYIPVADDFARKITENVVKD
tr|L8LLH3|L8LLH3_9CHRO           FFAKLHGNFQQAAA--EGKIVTCFVIQSLIIEAFAIAAYHLYIPVADPFARKITENVVKD
tr|K9Z5G1|K9Z5G1_CYAAP           YFAELHSNFQKAKE--EGKIVTCLLIQALIIEAFAIAAYNIYIPVADPFARKITEGVVKD
tr|I4FNH9|I4FNH9_MICAE           FFSSLHENFQIAYA--EGKVVTCLLIQSLIIEAFAIAAYNIYIPVADPFARKITEGVVKD
tr|I4I7T6|I4I7T6_9CHRO           FFSSLHENFQIAYA--EGKVVTCLLIQSLIIEAFAIAAYNIYIPVADPFARKITEGVVKD
tr|I4HKI6|I4HKI6_MICAE           FFSSLHNNFQIAYA--EGKVVTCLLIQSLIIEAFAIAAYNIYIPVADPFARKITEGVVKD
tr|B0JY93|B0JY93_MICAN           FFSSLHNNFQIAYA--EGKVVTCLLIQSLIIEAFAIAAYNIYIPVADPFARKITEGVVKD
tr|I4GYG9|I4GYG9_MICAE           FFSSLHENFQIAYA--EGKVVTCLLIQSLIIEAFAIAAYNIYIPVADPFARKITESVVKD
tr|I4IM50|I4IM50_MICAE           FFSSLHDNFQIAYA--EGKVVTCLLIQSLIIEAFAIAAYNIYIPVADPFARKITESVVKD
tr|I4FD05|I4FD05_MICAE           FFSSLHDNFQIAYA--EGKVVTCLLIQSLIIEAFAIAAYNIYIPVADPFARKITESVVKD
tr|L7E3D3|L7E3D3_MICAE           FFSSLHDNFQIAYA--EGKVVTCLLIQSLIIEAFAIAAYNIYIPVADPFARKITESVVKD
tr|L8NPI8|L8NPI8_MICAE           FFSSLHNNFQIAYA--EGKVVTCLLIQSLIIEAFAIAAYNIYIPVADPFARKITESVVKD
tr|A8YJD6|A8YJD6_MICAE           FFSSLHNNFQIAYA--EGKVVTCLLIQSLIIEAFAIAAYNIYIPVADPFARKITESVVKD
tr|S3JGS6|S3JGS6_MICAE           FFSSLHNNFQIAYA--EGKIVTCLLIQSLIIEAFAIAAYNIYIPVADPFARKITESVVKD
tr|I4GKL8|I4GKL8_MICAE           FFSSLHNNFQIAYA--EGKIVTCLLIQSLIIEAFAIAAYNIYIPVADPFARKITESVVKD
tr|I4HLI6|I4HLI6_MICAE           FFSSLHNNFQIAYA--EGKIVTCLLIQSLIIEAFAIAAYNIYIPVADPFARKITESVVKD
tr|I4FY74|I4FY74_MICAE           FFSSLHNNFQIAYA--EGKVVTCLLIQSLIIEAFAIAAYNIYIPVADPFARKITEGVVKD
tr|I4H417|I4H417_MICAE           FFSSLHNNFQIAYA--EGKVVTCLLIQSLIIEAFAIAAYNIYIPVADPFARKITEGVVKD
tr|M1LIQ1|M1LIQ1_9SYNC           FFAGLHGNFQHAFS--EGKVVTCLLIQALIIEAFAIAAYNIYIPVADDFARKITEGVVKD
tr|H0NYN3|H0NYN3_9SYNC           FFAGLHGNFQHAFS--EGKVVTCLLIQALIIEAFAIAAYNIYIPVADDFARKITEGVVKD
tr|H0PB35|H0PB35_9SYNC           FFAGLHGNFQHAFS--EGKVVTCLLIQALIIEAFAIAAYNIYIPVADDFARKITEGVVKD
tr|H0PG18|H0PG18_9SYNC           FFAGLHGNFQHAFS--EGKVVTCLLIQALIIEAFAIAAYNIYIPVADDFARKITEGVVKD
tr|F7US88|F7US88_SYNYG           FFAGLHGNFQHAFS--EGKVVTCLLIQALIIEAFAIAAYNIYIPVADDFARKITEGVVKD
sp|Q55688|ALDEC_SYNY3            FFAGLHGNFQHAFS--EGKVVTCLLIQALIIEAFAIAAYNIYIPVADDFARKITEGVVKD
tr|D3EPA5|D3EPA5_ATETH           FFSQLHENFQIAKN--EKKVVTCLLIQALIIEAFAIAAYNIYIPVADPFARKITENVVKD
tr|B7JUH1|B7JUH1_CYAP8           FFSSLHGNFQKAKA--EGKIVTCLLIQSLIIEAFAIAAYNIYIPVADPFARKITEGVVKD
tr|C7QUV0|C7QUV0_CYAP0           FFSSLHGNFQKAKA--EGKIVTCLLIQSLIIEAFAIAAYNIYIPVADPFARKITEGVVKD
tr|G5J0P0|G5J0P0_CROWT           FFSQLHGNFQTAKA--EGKIVTCLLIQSLIIEAFAIAAYNIYIPVADPFARKITENVVKD
tr|T2JF13|T2JF13_CROWT           FFSQLHGNFQTAKA--EGKIVTCLLIQSLIIEAFAIAAYNIYIPVADPFARKITENVVKD
tr|Q4C7T3|Q4C7T3_CROWT           FFSQLHGNFQTAKA--EGKIVTCLLIQSLIIEAFAIAAYNIYIPVADPFARKITENVVKD
tr|T2IH71|T2IH71_CROWT           FFSQLHGNFQTAKA--EGKIVTCLLIQSLIIEAFAIAAYNIYIPVADPFARKITENVVKD
tr|A3INE1|A3INE1_9CHRO           FFSQLHNNFQTAKA--EGKIVTCLLIQSLIIEAFAIAAYNIYIPVADPFARKITEGVVKD
tr|B1WR71|B1WR71_CYAA5           FFAELHGNFQKAKA--EGKIVTCLLIQSLIIEAFAIAAYNIYIPVADPFARKITEGVVKD
tr|U5DFA3|U5DFA3_9CHRO           YFARLHGNFQQALA--DGDVVACFLIQSLIIECFAIAAYNIYIPVADPFARKITEGVVKD
tr|L8N0G8|L8N0G8_9CYAN           FFSELHDNFQKAFA--IGDIVTCLLIQSLIIECFAIAAYNIYIPVADPFARKITEGVVKD
tr|K9SPY2|K9SPY2_9CYAN           FFAQLHGNFQTAAA--EGDVVTCLLIQSLIIECFAIAAYNIYIPVADDFARKITEGVVKD
tr|K9SZC3|K9SZC3_9SYNE           FFNALHGNFQKAFA--ITDVVTCLLIQSLIIECFAIAAYNIYIPVADDFARKITEGVVKD
tr|B4WJ48|B4WJ48_9SYNE           FFAQLHDNFQTALA--EGKIVTCLLIQSLIIETFAISAYNIYIPVADDFARKITEGVVKD
tr|U9WCI1|U9WCI1_9CYAN           FFSKLHDNFRQARD--EGKIVTCLLIQSLIIETFAISAYNIYIPVADDFARKITEGVVKD
tr|W5U411|W5U411_9CYAN           FFAKLHDNFKQAQD--EGKIVTCLLIQSLIIETFAISAYNIYIPVADDFARKITEGVVKD
tr|K9F0G4|K9F0G4_9CYAN           FFAKLHDNFKQARD--EGKVVTCLLIQSLIIETFAISAYNIYIPVADDFARKITEGVVKD
tr|K9ULL9|K9ULL9_9CHRO           FFAQLHENFQVAAA--AGNIVTCLLIQSLIIECFAIAAYNIYIPVADDFAKKITEAVVKD
tr|K8GFP8|K8GFP8_9CYAN           FFSSLHGNFQKAAA--EGRLVTCLLIQSLIIECFAIAAYNIYIPVADAFARKITEGVVKD
tr|D4TEY2|D4TEY2_9NOST           FFSPLHNNFQIAAQQPKPKVVTCLLIQSLIIECFAIAAYNIYIPVADPFARKITEGVVKE
tr|D4TRY1|D4TRY1_9NOST           FFSPLHNNFQIAAQQG--KVVTCLLIQSLIIECFAIAAYNIYIPVADPFARKITEGVVKE
tr|K9V615|K9V615_9CYAN           FFAGLHKNFQDAAA--EGKVVTCLLIQSLIIECFAIAAYNIYIPVADDFARKITEGVIKE
tr|K9PTP6|K9PTP6_9CYAN           FFSGLHGNFQAAAA--AGKVVTCLLIQSLIIECFAIAAYNIYIPVADDFARKITEGVVKD
tr|K7WTY7|K7WTY7_9NOST           FFLGLHDNFQTAAE--EGKVVTCLLIQSLIIECFAIAAYNIYIPVADDFARKITEGVVKE
tr|K9WRL8|K9WRL8_9NOST           FFLGLHQNFQTAAA--AGKVVTCLLIQSLIIECFAIAAYNIYIPVADEFARKITEGVVKE
tr|K9ZJT3|K9ZJT3_ANACC           FFSGLHKNFQTAAA--EGKVVTCLLIQALIIECFAIAAYNIYIPVADDFARKITEGVVKE
tr|D7E2W1|D7E2W1_NOSA0           FFSGLHQNFQTAAA--AGNVVTCLLIQSLIIECFAIAAYNIYIPVADDFARKITEGVVKE
tr|Q112R8|Q112R8_TRIEI           FFEPLHENFQTAAA--TGNVVTCLLIQSLIIECFAIAAYNIYIPVADPFARKITESVVKD
sp|Q54764|ALDEC_SYNE7            FFERLHENFKAAAA--EGKVVTCLLIQSLIIECFAIAAYNIYIPVADAFARKITEGVVRD
tr|Q8KPT4|Q8KPT4_SYNE7           FFERLHENFKAAAA--EGKVVTCLLIQSLIIECFAIAAYNIYIPVADAFARKITEGVVRD
tr|Q5N627|Q5N627_SYNP6           FFERLHENFKAAAA--EGKVVTCLLIQSLIIECFAIAAYNIYIPVADAFARKITEGVVRD
tr|K9RFH5|K9RFH5_9CYAN           FFAQLHQNFQEAAA--EGKVVTCLLIQSLIIECFAIAAYHIFIPVADDFSRKITEGVVKD
tr|K9TXF6|K9TXF6_9CYAN           FFSGLHQNFQQAAS--AGQVVTCLLIQSLIIECFAIAAYNIYIPCADDFARKITEGVVKD
tr|K9X9I4|K9X9I4_9CHRO           FFAKLHQNFQKAAE--SGNVVTCLLIQSLIIECFAIAAYNIYIPVADDFARKITEGVVKD
tr|A0Z9X5|A0Z9X5_NODSP           FFSGLHENFQKAAA--EGQVVTCLLIQSLIIECFAIAAYNIYIPVADDFARKITEGVVKD
tr|Q3MA38|Q3MA38_ANAVT           FFAGLHGNFQKAAA--EGKVVTCLLIQSLIIECFAIAAYNIYIPVADDFARKITEGVVKD
```

```
                            ..90.......100  .......110.......120.......130.......140....
                             *  *   *             *:::* :::* **::**. ::  :* ::: :*  *: :
tr|Q8YLL5|Q8YLL5_NOSS1      FFAGLHGNFQKAAA--EGKIVTCLLIQSLIIECFAIAAYNIYIPVADDFARKITEGVVKD
tr|K9QXA2|K9QXA2_NOSS7      FFAELHSNFQTAAA--EGKVVTCLLIQSLIIECFAIAAYNIYIPVADDFARKITEGVVKD
tr|M1X0T0|M1X0T0_9NOST      FFADLHHNFHTASA--EGKIVTCLLIQSLIIECFAIAAYNIYIPVADNFARKITENVVKD
tr|M1WVU9|M1WVU9_9NOST      FFADLHQNFHTASA--EGKIVTCLLIQSLIIECFAIAAYNIYIPVADNFARKITENVVKD
tr|K9S7C1|K9S7C1_9CYAN      FFADLHRNFQEAAA--QGKVVTCLLIQSLIIECFAIAAYNIYIPVADDFARKVTEGVVKD
tr|K9W4T2|K9W4T2_9CYAN      FFSRLHENFKVAAE--AGEIVTCLLIQSLIIECFAIAAYNIYIPVADDFARKITEGVVKD
tr|G6FV22|G6FV22_9CYAN      FFAQLHHNFQVAAQ--EGKIVTCLLIQSLIIECFAIAAYNIYIPVADDFARKITEGVVKD
tr|W5U379|W5U379_9CYAN      FFAQLHQNFQIAAQ--EGKIVTCLLIQSLIIECFAIAAYNIYIPVADDFARKITEGVVKD
tr|W5U417|W5U417_9CYAN      FFAQLHQNFQIAAQ--EGKIVTCLLIQSLIIECFAIAAYNIYIPVADDFARKITEGVVKD
tr|K9QA48|K9QA48_9NOSO      FFAPLHDNFKAGAA--ENKVVTCLLIQSLIIECFAIAAYNCYIPFADDFARKITEGVVKD
tr|G9HTG5|G9HTG5_9NOSO      FFAPLHDNFKAAAA--TGNVVTCLLIQSLIIECFAIAAYNIYIPVADDFARKITEGVVKD
tr|H2DDT7|H2DDT7_NOSS6      FFAPLHDNFKAAAA--TGNVVTCLLIQSLIIECFAIAAYNIYIPVADDFARKITEGVVKD
tr|K9YV86|K9YV86_DACSA      FFAELHQNFQDALA--EEKVVTCLLIQSLIIECFAISAYNIYIPVADPFARKITEGVVKD
tr|K9Y9U4|K9Y9U4_HALP7      FFAQLHQNFQDALA--EGKIVTCLLIQALIIECFAISAYNIYIPVADPFARKITEGVVKD
tr|W5U332|W5U332_PLAAG      FFSSLHQNFKDAAT--EGKVVTCLLIQSLIIECFAIAAYNIYIPVADDFARKITERVVKD
tr|F5UGX9|F5UGX9_9CYAN      FFSDLHRNFQTAAA--EGQIVTCLLIQSLIIECFAIAAYNIYIPVADDFARKITEGVVKE
tr|K9VE50|K9VE50_9CYAN      FFSELHRNFQTAAA--QGQIVTCLLIQSLIIECFAIAAYNIYIPVADDFARKITEGVVKE
tr|D8FVZ2|D8FVZ2_9CYAN      FFAALHSNFQEAAA--AGKVVTCLVIQALIIECFAIAAYNIYIPVADDFARKITEGVVKD
tr|B4VSW8|B4VSW8_9CYAN      YFSDLHQNFQTAAA--SGNIVTCLLIQSLIIECFAIAAYNIYIPVADPFARKITEGVVKD
tr|K9WII2|K9WII2_9CYAN      YFSQLHGNFQTAAA--TGNVVTCLLIQSLIIECFAIAAYNIYIPVADDFARKITEGVVKD
tr|K9TGS5|K9TGS5_9CYAN      FFSGLHQNFQTAAA--EGKVVTCLLIQALIIESFAISAYNIYIPVADPFARKITEGVVKD
tr|B5W0S5|B5W0S5_ARTMA      FFAKLHGNFQKAAA--EGKLVTCLLIQSLIIESFAIAAYNIYIPVADPFARKITEGVVKD
tr|K1WZT6|K1WZT6_ARTPT      FFAKLHGNFQKAAA--EGKLVTCLLIQSLIIESFAIAAYNIYIPVADPFARKITEGVVKD
tr|W6SNU7|W6SNU7_9CYAN      FFAKLHGNFQKAAA--EGKLVTCLLIQSLIIESFAIAAYNIYIPVADPFARKITEGVVKD
tr|H1WAD2|H1WAD2_9CYAN      FFAKLHGNFQKAAA--EGKLVTCLLIQSLIIESFAIAAYNIYIPVADPFARKITEGVVKD
tr|K6DQI0|K6DQI0_ARTPT      FFAQLHGNFQKAAA--EGKLVTCLLIQSLIIESFAIAAYNIYIPVADPFARKITEGVVKD
tr|D5A1Z8|D5A1Z8_ARTPN      FFAQLHGNFQKAAA--EGKLVTCLLIQSLIIESFAIAAYNIYIPVADPFARKITEGVVKD
tr|U7QM55|U7QM55_9CYAN      FFSQLHQNFQDAAA--EGKVVTCLLIQSLIIESFAIAAYNIYIPVADPFARKITEGVVQD
tr|A0YKK4|A0YKK4_LYNSP      FFSQLHQNFKDAAA--QGKVVTCLLIQSLIIESFAIAAYNIYIPVADPFARKITEGVVDD
tr|A3Z6M0|A3Z6M0_9SYNE      LFQPLHDLFKRHDH--DGNRAECLVIQGLIVECFAVAAYRHYLPVADAYARPITAAVMND
tr|B5ILL6|B5ILL6_9CHRO      FFAPLHGNFQRALA--EGKVTTCLLIQAILIEAFAISAYHIYIPVADPFARRITEGVVKD
tr|A3YYU8|A3YYU8_9SYNE      FFSPLHGNFQAALA--EGKVVTCLLIQAILIEAFAISAYHIYIPVADPFARKITEGVVKD
tr|A2C7S0|A2C7S0_PROM3      FFAPLRDNFQTALE--QGKTPTCLLIQALLIEAFAISAYHTYIPVSDPFARKITEGVVKD
sp|Q7V6D4|ALDEC_PROMM       FFAPLRDNFQTALG--QGKTPTCLLIQALLIEAFAISAYHTYIPVSDPFARKITEGVVKD
tr|A5GUD0|A5GUD0_SYNR3      FFSDLHGNFRSALE--ENKVVTCLVIQALMIEAFAIAAYHIYIPVADPFARKITEGVVKD
tr|K9P3X5|K9P3X5_CYAGP      FFSPLRNNFQAALK--EGKVVTCLLIQALLIEAFAISAYHIYIPVADPFARKITEGVVKD
tr|Q0I8M9|Q0I8M9_SYNS3      FFAPLHGNFQSALK--EGKVVTCLLIQALLIEAFAISAYHIYIPVADPFARKITEGVVKD
tr|G4FIH8|G4FIH8_9SYNE      FFAPLHGNFQSALK--EGKVVTCLLIQALLIEAFAISAYHIYIPVADPFARKITEGVVKD
tr|Q05RJ5|Q05RJ5_9SYNE      FFAPLHGNFQKAMK--EGKVVTCLLIQALLIEAFAISAYHIYIPVADPFARKITEGVVKD
tr|A5GJG5|A5GJG5_SYNPW      FFGPLHGNFQTALK--EGKVVTCLLIQALLIEAFAISAYHIYIPVADPFARKITEGVVKD
tr|A4CS34|A4CS34_SYNPV      FFAPLHGNFQTALK--DGKVVTCLLIQALLIEAFAISAYHIYIPVADPFARKITEGVVKD
tr|A3Z5H6|A3Z5H6_9SYNE      FFAPLHGNFQAALQ--EGKVVTCLLIQALLIEAFAISAYHIYIPVADPFARKITEGVVKD
tr|Q060S1|Q060S1_9SYNE      FFGPLHGNFQVALK--EGKVVTCLLIQALLIEAFAISAYHIYIPVADPFARKITEGVVKD
tr|Q3AV61|Q3AV61_SYNS9      FFGPLHGNFQVALK--EGKVVTCLLIQALLIEAFAISAYHIYIPVADPFARKITEGVVKD
tr|Q0QKQ4|Q0QKQ4_9SYNE      FFEPLHGNFQSALK--EGKVVTCLLIQALLIEAFAISAYHIYIPVADPFARKITEGVVKD
tr|Q0QM11|Q0QM11_9SYNE      FFEPLHRNFQSALQ--EGKVVTCLLIQALLIEAFAISAYHIYIPVADPFARKITEGVVKD
tr|Q3ALN2|Q3ALN2_SYNSC      FFEPLHGNFQAALK--EGKVVTCLLIQALLIEAFAISAYHIYIPVADPFARKITEGVVKD
tr|W0GVR9|W0GVR9_9SYNE      FFEPLHGNFQAAFK--EGKVVTCLLIQALLIEAFAISAYHIYIPVADPFARKITEGVVKD
tr|Q0QKF3|Q0QKF3_9SYNE      FFEPLHGNFQAALK--EGKVVTCLLIQALLIEAFAISAYHIYIPVADPFARKITEGVVKD
tr|Q7U5G9|Q7U5G9_SYNPX      FFEPLHGNFQAALK--EGKVVTCLLIQALLIEAFAISAYHIYIPVADPFARKITEGVVKD
tr|Q0QKK1|Q0QKK1_9SYNE      FFEPLHGNFQAALK--EGKVVTCLLIQALLIEAFAISAYHIYIPVADPFARKITEGVVKD
tr|A2C0Z0|A2C0Z0_PROM1      FFSKLHNNFLEAFK--VGDTTTCLLIQCILIESFAISAYHVYIRVADPFAKRITEGVVQD
tr|Q46GP5|Q46GP5_PROMT      FFSKLHNNFLEAFK--VGDTTTCLLIQCILIESFAISAYHVYIRVADPFAKRITEGVVQD
tr|A8G3P7|A8G3P7_PROM2      FFSKLHGNFQIALK--DGNLTTCLLIQAILIEAFAISAYHVYIRVADPFAKKITQGVVND
tr|A2BVJ4|A2BVJ4_PROM5      FFSKLHGNFQAALK--NESLTTCLLIQAILIEAFAISAYHVYIRVADPFAKKITQGVVND
tr|Q7V2E8|Q7V2E8_PROMP      FFSKLHGNFQVALS--NGKTTTCLLIQAILIEAFAISAYHVYIRVADPFAKKITQGVVKD
tr|Q31C02|Q31C02_PROM9      FFSKLHGNFQIALE--KGNLTTCLLIQAILIEAFAISAYNVYIRVADPFAKKITEGVVKD
tr|A2BQ13|A2BQ13_PROMS      FFSKLHGNFQVALE--KGNLTTCLLIQAILIEAFAISAYNVYIRVADPFAKKITEGVVKD
tr|A3PBQ6|A3PBQ6_PROM0      FFSKLHGNFQVALK--KGNVTTCLLIQAILIEAFAISAYNVYIRVADPFAKKITEGVVKD
tr|A9BEF6|A9BEF6_PROM4      FFAPLHGNFQKALE--NGKITTCFLIQAILIEAFAISAYHVYIRVADPFAKKITEGVVKD
tr|Q7VD51|Q7VD51_PROMA      FFSPLHGNFQKAFK--EENLTTCFLIQAILIEAFAISAYHVYIRVADPFAKKITENVVKD
tr|B8HLW2|B8HLW2_CYAP4      FFAELHQIFASAAN--AGQVATCLVVQALIIECFAIAAYNTYLPVADEFARKVTASVVQD
tr|U9VZT6|U9VZT6_9CYAN      FFADLCQAFQTSAT--DRKVATCLLIQSLVIECFAISAYNVYIPVADEFAQKVTKGVVAD
tr|K9EQR0|K9EQR0_9CYAN      FFADLCQAFQTSAT--DRKVATCLLIQSLIIECFAIAAYNVYIPVADEFAQKVTKGVVAD
```

```
                            ...150.......160.......170.......180.......190.......200....
                            *  * .:.: **   :   :  :  .    :*    :*  :  :   : *     :  *
sp|B2J1M1|ALDEC_NOSP7       EYSHLNFGEVWLKEHFAESKAELELANRQNLPIVWKMLNQVEGDAHTMAMEKDALVEDFM
tr|U5QDK0|U5QDK0_9CYAN      EYTHLNFGQQWLKDHFEAVRPGIEQANAQNLPIVWRMLGEVEDDAATLQMDKEAIVEDFL
tr|Q7NGM3|Q7NGM3_GLOVI      EYTHLNFGQQWLKERFVTVREGIERANAQNLPIVWRMLNAVEADTEVLQMDKEAIVEDFM
tr|Q2JJ13|Q2JJ13_SYNJB      EYLHLNFGERWLGEHFGEVKGQIEAANAQNLPLVWQMLQQVDQDVEAIYMDREAIVEDFM
tr|Q2JX62|Q2JX62_SYNJA      EYLHLNFGERWLGEHFAEVKAQIEAANAQNLPLVRQMLQQVEADVEAIYMDREAIVEDFM
tr|K9RTZ6|K9RTZ6_SYNP3      EYSHLNFGEVWLQENFEAVKAEVETANKDNLPLVWRMLNEVEKDAKVLGMDKDALVEDFM
tr|Q8DJB4|Q8DJB4_THEEB      EYSHLNFGEIWLKEHFESVKGELEEANRANLPLVWKMLNQVEADAKVLGMEKDALVEDFM
tr|V5V382|V5V382_9CHRO      EYSHLNFGEIWLKENFESVKAELEEANRANLPLVWKMLNQVEADAKVLGMEKDALVEDFM
tr|B0C9L0|B0C9L0_ACAM1      EYQHLNYGEEWLKAHFDDVKAEIQEANRKNLPIVWRMLNEVDKDAAVLGMEKEALVEDFM
tr|B8HSZ3|B8HSZ3_CYAP4      EYTHLNFGEEWLKANFDSQREEVEAANRENLPIVWRMLNQVETDAHVLGMEKEALVESFM
tr|L8LLH3|L8LLH3_9CHRO      EYTHLNFGEEWLKGNFASAKAELEEANRENLPIVWQMLNEVAEDAAILAMEKDALVEDFM
tr|K9Z5G1|K9Z5G1_CYAAP      EYLHLNFGEEWLKANFEASKAELEEANRQNLPIIWRMLNEVEKDAKTLGMPKEELIEDFM
tr|I4FNH9|I4FNH9_MICAE      EYLHLNFGEEWLKANFETAKKELEAANRANLPIVWRMLNQVEDDARVLGMEKEALVEDFM
tr|I4I7T6|I4I7T6_9CHRO      EYLHLNFGEEWLKANFETAKKELEAANRANLPIVWRMLNQVEDDARVLGMEKEALVEDFM
tr|I4HKI6|I4HKI6_MICAE      EYLHLNFGEEWLKANFETAKEELEAANRANLPIVWRMLNQVENDARVLGMEKEALVEDFM
tr|B0JY93|B0JY93_MICAN      EYLHLNFGEEWLKANFETAKEELEAANRANLPIVWRMLNQVENDARVLGMEKEALVEDFM
tr|I4GYG9|I4GYG9_MICAE      EYLHLNFGEEWLKANFETAKEELEAANRANLPIVWRMLNQVEDDARVLGMEKEALVEDFM
tr|I4IM50|I4IM50_MICAE      EYLHLNFGEEWLKANFETAKEELEAANRANLPIVWRMLNQVEDDARVLAMEKEALVEDFM
tr|I4FD05|I4FD05_MICAE      EYLHLNFGEEWLKANFETAKEELEAANRANIPIVWRMLNQVEDDARVLAMEKEALVEDFM
tr|L7E3D3|L7E3D3_MICAE      EYLHLNFGEEWLKANFETAKEELEAANRANLPIVWRMLNQVEDDARVLAMEKEALVEDFM
tr|L8NPI8|L8NPI8_MICAE      EYLHLNFGEEWLKANFETAKEELEAANRANLPIVWRMLNQVEDDARVLAMEKEALVEDFM
tr|A8YJD6|A8YJD6_MICAE      EYLHLNFGEEWLKANFETAKEELEAANRANLPIVWRMLNQVEDDARVLAMEKEALVEDFM
tr|S3JGS6|S3JGS6_MICAE      EYLHLNFGEEWLKANFETAKEELEAANRANLPIVWKMLNQVEDDARVLAMEKEALVEDFM
tr|I4GKL8|I4GKL8_MICAE      EYLHLNFGEEWLKANFETAKEELEAANRANLPIVWRMLNQVEDDARVLAMEKEALVEDFM
tr|I4HLI6|I4HLI6_MICAE      EYLHLNFGEEWLKANFETAKEELEAANRANLPIVWRMLNQVEDDARVLAMEKEALVEDFM
tr|I4FY74|I4FY74_MICAE      EYLHLNFGEEWLKANFETAKEELEAANRANLPIVWKMLNQVEDDARVLAMEKEALVEDFM
tr|I4H417|I4H417_MICAE      EYLHLNFGEEWLKANFETAKDELEAANRANLPIVWRMLNQVEDDARVLAMEKEALVEDFM
tr|M1LIQ1|M1LIQ1_9SYNC      EYTHLNYGEEWLKANFATAKEELEQANKENLPLVWKMLNQVQGDAKVLGMEKEALVEDFM
tr|H0NYN3|H0NYN3_9SYNC      EYTHLNYGEEWLKANFATAKEELEQANKENLPLVWKMLNQVQGDAKVLGMEKEALVEDFM
tr|H0PB35|H0PB35_9SYNC      EYTHLNYGEEWLKANFATAKEELEQANKENLPLVWKMLNQVQGDAKVLGMEKEALVEDFM
tr|H0PG18|H0PG18_9SYNC      EYTHLNYGEEWLKANFATAKEELEQANKENLPLVWKMLNQVQGDAKVLGMEKEALVEDFM
tr|F7US88|F7US88_SYNYG      EYTHLNYGEEWLKANFATAKEELEQANKENLPLVWKMLNQVQGDAKVLGMEKEALVEDFM
sp|Q55688|ALDEC_SYNY3       EYTHLNYGEEWLKANFATAKEELEQANKENLPLVWKMLNQVQGDAKVLGMEKEALVEDFM
tr|D3EPA5|D3EPA5_ATETH      EYKHLNFGEVWLGENFESSKIELEEANKTNLPIVWKMLNEVEQDASILGMEKEALVEDFM
tr|B7JUH1|B7JUH1_CYAP8      EYTHLNFGEVWLQEHFEESKAELEEANKANLPIVWEMLNQVEGDAKVLGMEKEALVEDFM
tr|C7QUV0|C7QUV0_CYAP0      EYTHLNFGEVWLQEHFEESKAELEEANKANLPIVWEMLNQVEGDAKVLGMEKEALVEDFM
tr|G5J0P0|G5J0P0_CROWT      EYSHLNFGEVWLKENFEASKAELEQANKENLPIVWQMLNEVEDDAEILGMEKEALVEDFM
tr|T2JF13|T2JF13_CROWT      EYSHLNFGEVWLKENFEASKAELEQANKENLPIVWQMLNEVEDDAEILGMEKEALVEDFM
tr|Q4C7T3|Q4C7T3_CROWT      EYSHLNFGEVWLKENFEASKAELEQANKENLPIVWQMLNEVEDDAEILGMEKEALVEDFM
tr|T2IH71|T2IH71_CROWT      EYSHLNFGEVWLKENFEASKAELEQANKENLPIVWQMLNEVEDDAEILGMEKEALVEDFM
tr|A3INE1|A3INE1_9CHRO      EYTHLNFGEIWLKEHFEASKAELEEANKKNLPIVWQMLNQVEKDAEVLGMEKEALVEDFM
tr|B1WR71|B1WR71_CYAA5      EYTHLNFGEVWLKEHFEASKAELEDANKENLPLVWQMLNQVEKDAEVLGMEKEALVEDFM
tr|U5DFA3|U5DFA3_9CHRO      EYLHLNFGEVWLREHFEDVKERLLEANKQNLPIVWQMLNQVEDDAAVLGMGKEELIEDFM
tr|L8N0G8|L8N0G8_9CYAN      EYLHLNFGEEWLQDHFENSRATLEQANRQNLPLVWKMLNQVETDAKVLGMEKEALVEDFM
tr|K9SPY2|K9SPY2_9CYAN      EYLHLNFGEEWLKANFESAKAGLEKANRQNLPIVWKMLNRVEKDAKVLGMEKEALVEDFM
tr|K9SZC3|K9SZC3_9SYNE      EYLHLNFGEVWLQENFETAKNQLEIANRQNLPLVWKMLNQVAKDAKVLGMEKDALVEDFM
tr|B4WJ48|B4WJ48_9SYNE      EYMHLNFGEEWLKANFEASKAELETANRANLPLIWKMLNQVEEDAAVLGMEKDALIEDFM
tr|U9WCI1|U9WCI1_9CYAN      EYMHLNFGEEWLKANFETAKAELEEANRQNLPLVWQMLNEVADDAAVLGMEKEALVEDFM
tr|W5U411|W5U411_9CYAN      EYMHLNFGEEWLKANFDSAKEELEEANRQNLPLVWQMLNQVADDAAILGMEKDALVEDFM
tr|K9F0G4|K9F0G4_9CYAN      EYMHLNFGEEWLKANFDSAKKELEEANRQNLPLVWQMLNQVADDAAILGMEKDALVEDFM
tr|K9ULL9|K9ULL9_9CHRO      EYMHLNFGEVWLHDNFEASKAELQTANRQNLPLVWKMLNNVAKDAKVLGMEKDALVEDFM
tr|K8GFP8|K8GFP8_9CYAN      EYSHLNFGEEWLKANFEQSKAELEAANRENLPLVWQMLNQVEEDARVLGMEKDALVEDFM
tr|D4TEY2|D4TEY2_9NOST      EYTHLNFGEVWLKEKFEASKEELEVANRENLPIVWQMLNQVAEDAKVLGMEKDALVEDFM
tr|D4TRY1|D4TRY1_9NOST      EYTHLNFGEVWLKEKFEASKEELEVANRENLPIVWQMLNQVAEDAKVLGMEKDALVEDFM
tr|K9V615|K9V615_9CYAN      EYMHLNFGEVWLQANFETAKAELEEANRQNLPLVWQMLNQVADDAEVLAMEKDALVEDFM
tr|K9PTP6|K9PTP6_9CYAN      EYSHLKFGEVWLQEHFTEAKAELEEANRQNLPIVWKMLNQVADDAKILAMEKDALVEDFM
tr|K7WTY7|K7WTY7_9NOST      EYSHLNFGEVWLQENFTESQAELEAANRQNLPIVWKMLNEVADDAKVLAMEKDALVEDFM
tr|K9WRL8|K9WRL8_9NOST      EYSHLNFGEVWLQENFAESKAELEEANRHNLPIVWKMLNQVADDAQVLAMEKEALVEDFM
tr|K9ZJT3|K9ZJT3_ANACC      EYSHLNFGEVWLQENFAESKAELETANRQNLPLVWKMLNQVADDAHVLAMEKEALVEDFM
tr|D7E2W1|D7E2W1_NOSA0      EYSHLNFGEVWLKEHFAESKAELDDANRQNLPIVWQMLNQVADDARVLAMEKEALVEDFM
tr|Q112R8|Q112R8_TRIEI      EYSHLNFGEVWLKEYFEDSKQELQKANRQNLPLVWKMLNQVEKDAKTLEMEKEALIEDFM
sp|Q54764|ALDEC_SYNE7       EYLHRNFGEEWLKANFDASKAELEEANRQNLPLVWLMLNEVADDARELGMERESLVEDFM
tr|Q8KPT4|Q8KPT4_SYNE7      EYLHRNFGEEWLKANFDASKAELEEANRQNLPLVWLMLNEVADDARELGMERESLVEDFM
tr|Q5N627|Q5N627_SYNP6      EYLHRNFGEEWLKANFDASKAELEEANRQNLPLVWLMLNEVADDARELGMERESLVEDFM
tr|K9RFH5|K9RFH5_9CYAN      EYTHLNFGEVWLKEHFEESKAELEEANAQNLPIVWKMLNEVETDAEVMAMPKDALVEDFM
tr|K9TXF6|K9TXF6_9CYAN      EYTHLNFGEVWLKDNFEQSKAELEAANRQNLPIVWQMLNKVEDDARVLGMEKDALVEDFM
tr|K9X9I4|K9X9I4_9CHRO      EYSHLNFGEVWLKEHFEASKAELEQANRQNLPIVWQMLNAVENDAHTLAMEKEALVEDFM
tr|A0Z9X5|A0Z9X5_NODSP      EYSHLNFGEVWLKENFAQSKAELEAANRQNLPIVWKMLNEVENDAHVLAMEKEALVEDFM
tr|Q3MA38|Q3MA38_ANAVT      EYSHLNFGEVWLQKNFAQSKAELEEANRHNLPIVWKMLNQVADDAAVLAMEKEALVEDFM
```

```
                                ...150.......160.......170.......180.......190.......200....
                                *  * .:.: **   :   :  :  .    :*    :*  :  :   : *     :  *
tr|Q8YLL5|Q8YLL5_NOSS1          EYSHLNFGEVWLQKNFAQSKAELEEANRHNLPIVWKMLNQVADDAAVLAMEKEALVEDFM
tr|K9QXA2|K9QXA2_NOSS7          EYSHLNFGEVWLKEHFAESKAELEAANRQNLPIVWRMLNQVEADAAILAMEKEALVEDFM
tr|M1X0T0|M1X0T0_9NOST          EYTHLNFGEVWLKENFEDSKAELEEANRQNLPIVWKMLSSVEEDAAVLAMDKEALVEDFM
tr|M1WVU9|M1WVU9_9NOST          EYTHLNFGEVWLKENFEDSKAELEEANRQNLPIVWKMLSSVEEDAAVLAMDKEALVEDFM
tr|K9S7C1|K9S7C1_9CYAN          EYSHLNFGEEWLKANFEASKAELEEANRQNLPLVWRMLNQVEQDAKTLAMEKDALVEDFM
tr|K9W4T2|K9W4T2_9CYAN          EYTHLNFGEVWLKEHFEESKAELETANRQNLPIVWQMLNQVAKDAGVLAMEKDALVEDFM
tr|G6FV22|G6FV22_9CYAN          EYSHLNFGEVWLKEHFEESKAELEEANRQNLPIVWQMLNQVADDAEVLAMEKEALVEDFM
tr|W5U379|W5U379_9CYAN          EYSHLNFGEVWLKEHFEESKAELEEANRQNLPIVWQMLNQVADDAEVLAMEKEALVEDFM
tr|W5U417|W5U417_9CYAN          EYSHLNFGEVWLKEHFEESKAELEEANRQNLPIVWQMLNQVADDAEVLAMEKEALVEDFM
tr|K9QA48|K9QA48_9NOSO          EYSHLNFGEVWLQQNFEASKIELEEANRQNLPIVWKMLNQVADDALVLGMEKDALVEDFM
tr|G9HTG5|G9HTG5_9NOSO          EYSHLNFGEVWLKANFEASKAELEEANRQNLPIVWKMLNQVAADAEVLAMEKEALVEDFM
tr|H2DDT7|H2DDT7_NOSS6          EYSHLNFGEVWLKANFEASKAELEEANRQNLPIVWKMLNQVAADAEVLAMEKEALVEDFM
tr|K9YV86|K9YV86_DACSA          EYTHLNYGEEWLKAHFDEVKDELEVANRQNLPIIWKMLNQVAADAKILGMEKDALVEDFM
tr|K9Y9U4|K9Y9U4_HALP7          EYTHLNYGEEWLKANFDSAKEELEEANRQNLPIVWKMLNQVAADANVLGMEKDALVEDFM
tr|W5U332|W5U332_PLAAG          EYMHLNFGEEWLKANFEASKTELETANRQNLPIIWRMLNQVADDAHILGMEKEALVEDFM
tr|F5UGX9|F5UGX9_9CYAN          EYSHLNFGEVWLQAHFEESKAELEAANRQNLPIIWKLLNAVADDARVLGMEKDALIEDFM
tr|K9VE50|K9VE50_9CYAN          EYSHLNFGEVWLQAHFEESKAELEAANRQNLPIIWKLLNAVADDARVLGMEKDALIEDFM
tr|D8FVZ2|D8FVZ2_9CYAN          EYNHLNFGEEWLKAHFEESKAEVDTANRQNLPIVWRMLNQVEDDARVLGMEKDALVEDFM
tr|B4VSW8|B4VSW8_9CYAN          EYMHLNFGEEWLKENFEASKTELEQANKQNLPLVWRMLNQVEKDAHILGMEKDALVEDFM
tr|K9WII2|K9WII2_9CYAN          EYMHLNFGEVWLKDHFEESKAELEQANRQNLPLVWRMLNQVEDDAHILGMEKDALVEDFM
tr|K9TGS5|K9TGS5_9CYAN          EYSHLNFGEKWLQAHFAESKTELEQANRQNLPIVWKMLNQVTDDAKVLEMEKEALVEDFM
tr|B5W0S5|B5W0S5_ARTMA          EYEHLNFGEEWLKAHFEESKAELEEANRQNLPLVWKMLNQVEKDASILGMEKEALIEDFM
tr|K1WZT6|K1WZT6_ARTPT          EYEHLNFGEEWLKAHFEESKAELEEANRQNLPLVWKMLNQVEKDASILGMEKEALIEDFM
tr|W6SNU7|W6SNU7_9CYAN          EYEHLNFGEEWLKAHFEESKAELEEANRQNLPLVWKMLNQVEKDASILGMEKEALIEDFM
tr|H1WAD2|H1WAD2_9CYAN          EYEHLNFGEEWLKAHFEESKAELEEANRQNLPLVWKMLNQVEKDASILGMEKEALIEDFM
tr|K6DQI0|K6DQI0_ARTPT          EYEHLNFGEEWLKAHFEESKAELEEANRQNLPLVWKMLNQVEKDASILGMEKEALIEDFM
tr|D5A1Z8|D5A1Z8_ARTPN          EYEHLNFGEEWLKAHFEESKAELEEANRQNLPLVWKMLNQVEKDASILGMEKEALIEDFM
tr|U7QM55|U7QM55_9CYAN          EYMHLNFGEEWLKAHFEESKAELEEANSQNLPIVWKMLNEVEKDAHILGMEKDALVEDFM
tr|A0YKK4|A0YKK4_LYNSP          EYMHLNFGEEWLKAHFEESKAELQEANSQNLPLVWKMLNEVENDAHILGMEKDALVEDFM
tr|A3Z6M0|A3Z6M0_9SYNE          ESEHLDYAETWLQRHFDQVKARVSAVVVEALPLTLAMLQSLAADMRQIGMDPVETLASFS
tr|B5ILL6|B5ILL6_9CHRO          EYTHLNYGQEWLKANLADVREELEQANRENLPLVRKMLEQVAGDAAVLQMDKEDLMADFL
tr|A3YYU8|A3YYU8_9SYNE          EYTHLNYGQEWLKANLESSRGELEQANRVNLPLVRKMLEQVAGDAAVLHMDQEDLMADFM
tr|A2C7S0|A2C7S0_PROM3          EYTHLNYGEAWLKANLESCREELLEANRENLPLIRRMLDQVAGDAAVLQMDKEDLIEDFL
sp|Q7V6D4|ALDEC_PROMM           EYTHLNYGEAWLKANLESCREELLEANRENLPLIRRMLDQVAGDAAVLQMDKEDLIEDFL
tr|A5GUD0|A5GUD0_SYNR3          EYTHLNYGQEWLKANFDSSRDEIIEANKANLPIIRRMLEEVADDAAELKMEKESLIEDFL
tr|K9P3X5|K9P3X5_CYAGP          EYTHLNYGQEWLKAHLEESRAELEQANRDNLPHVRRMLDRVAADAAVLHMEKEDLIEDFL
tr|Q0I8M9|Q0I8M9_SYNS3          EYTHLNYGQEWLKANFEASRDELMEANKVNLPLIRSMLEQVAADASVLHMEKEDLIEDFL
tr|G4FIH8|G4FIH8_9SYNE          EYTHLNYGQEWLKANFEASRDELMEANKVNLPLIRSMLEQVAADASVLHMEKEDLIEDFL
tr|Q05RJ5|Q05RJ5_9SYNE          EYTHLNYGQEWLKANFEASKDELMEANKVNLPLIRSMLEEVAKDAAVLHMEKEDLIEDFL
tr|A5GJG5|A5GJG5_SYNPW          EYTHLNYGQEWLKANFEASREELMEANKVNLPLIRSMLEQVAKDAAVLKMEKEDLIEDFL
tr|A4CS34|A4CS34_SYNPV          EYTHLNYGQEWLKANFDASREELMEANKVNLPLIRSMLEQVAEDAAVLKMEKEDLIEDFL
tr|A3Z5H6|A3Z5H6_9SYNE          EYTHLNYGQEWLKANFEASKDELMEANKANLPLIRSMLEQVAADAAVLQMEKEDLIEDFL
tr|Q060S1|Q060S1_9SYNE          EYTHLNYGQEWLKANFEASKDEMFAANKANLPLIRSMLEGVAADAAVLHMEKEDLIEDFL
tr|Q3AV61|Q3AV61_SYNS9          EYTHLNYGQEWLKANFEASKDEMFAANKANLPLIRSMLEGVAADAAVLHMEKEDLIEDFL
tr|Q0QKQ4|Q0QKQ4_9SYNE          EYTHLNYGQEWLKANFEASKEELFEANKANLPLIRSMLEDVAADAAVLHMEKEDLIEDFL
tr|Q0QM11|Q0QM11_9SYNE          EYTHLNYGQEWLKANFEASKDELFEANKANLPLIRSMLDDVAGDAAVLHMEKEDLIEDFL
tr|Q3ALN2|Q3ALN2_SYNSC          EYTHLNYGQEWLKANFEASKNELFEANKANLPLIRSMLEDVAADAAVLHMEKEDLIEDFL
tr|W0GVR9|W0GVR9_9SYNE          EYTHLNYGQEWLKANFEASKDELFEANKANLPLIRSMLEDVAADAAVLHMEKEDLIEDFL
tr|Q0QKF3|Q0QKF3_9SYNE          EYTHLNYGQEWLKANFEASKDELFEANKANLPLIRSMLEDVASDAAVLHMEKEDLIEDFL
tr|Q7U5G9|Q7U5G9_SYNPX          EYTHLNYGQEWLKANFEASKDELFEANKANLPLIRSMLEEVASDAAVLHMEKEDLIEDFL
tr|Q0QKK1|Q0QKK1_9SYNE          EYTHLNYGQEWLKANFEASKDELFEANKANLPLIRSMLEEVASDAAVLHMEKEDLIEDFL
tr|A2C0Z0|A2C0Z0_PROM1          EYLHLNYGQEWLKANLETVKKDLMRANKENLPLIKSMLDEVSNDAEVLHMDKEELMEEFM
tr|Q46GP5|Q46GP5_PROMT          EYLHLNYGQEWLKANLETVKKDLMRANKENLPLIKSMLDEVSNDAEVLHMDKEELMEEFM
tr|A8G3P7|A8G3P7_PROM2          EYLHLNYGEKWLKENLHTCKDELIAANKVNLPLIKKMLDQVAEDAATLSMDKEELMEEFM
tr|A2BVJ4|A2BVJ4_PROM5          EYLHLNYGEKWLKENLSTCKDELIAANKVNLPIIKKMLDQVADDAATLAMDKEELMEEFM
tr|Q7V2E8|Q7V2E8_PROMP          EYLHLNYGQEWLKENLATCKDELMEANKVNLPLIKKMLDQVSEDASVLAMDREELMEEFM
tr|Q31C02|Q31C02_PROM9          EYLHLNYGQEWLKKNLSTCKEELMEANKVNLPLIKKMLDEVAEDASVLAMDREELMEEFM
tr|A2BQ13|A2BQ13_PROMS          EYLHLNYGQEWLKENLSTCKDELMEANKVNLPLIKKMLDEVADDASVLAMDKEELMEEFM
tr|A3PBQ6|A3PBQ6_PROM0          EYLHLNYGQQWLKENLSTCKDELMEANKVNLPLIKKMLDEVADDASVLAMDREELMEEFM
tr|A9BEF6|A9BEF6_PROM4          EYLHLNYGQEWLKANLATCKDELIAANKENLPLINSMLDQVANDAQVLYMEKEELMEEFM
tr|Q7VD51|Q7VD51_PROMA          EYLHLNYGQQWLKANLDTCKEELMKANKENLPLIKSMLDQVADDACSLSMDKEELMEEFM
tr|B8HLW2|B8HLW2_CYAP4          EYSHLNFGEVWLQNAFEQCKDEIITANRLALPLIWKMLNQVTGELRILGMDKASLVEDFS
tr|U9VZT6|U9VZT6_9CYAN          EYHHLNFGEVWLKAHFETVKAELETANRQVLPLIWRMLNQVEADLQTVGMDKQTLIEAFL
tr|K9EQR0|K9EQR0_9CYAN          EYHHLNFGEVWLKAHFETVKAELETANRQVLPLIWQMLNQVDADLQTVGMDKQTLIESFL
```

```
                             ...210.......220.......230..
                               :  :*  :*:   :   :   .
sp|B2J1M1|ALDEC_NOSP7        IQYGEALSNIGFSTRDIMRLSAYGLIGA---
tr|U5QDK0|U5QDK0_9CYAN       IQYGEALSDIGFTTRDVMKMSARGLAAAPRA
tr|Q7NGM3|Q7NGM3_GLOVI       IAYGEALGDIGFSMRDVMKMSARGLASAPRQ
tr|Q2JJ13|Q2JJ13_SYNJB       IAYGEALANIGFSTREVMRLSAQGLRAA---
tr|Q2JX62|Q2JX62_SYNJA       IAYGEALASIGFNTREVMRLSAQGLRAA---
tr|K9RTZ6|K9RTZ6_SYNP3       IQYSGALENIGFTTREIMKMSMHGLVAA---
tr|Q8DJB4|Q8DJB4_THEEB       IQYSGALENIGFTTREIMKMSVYGLTGA---
tr|V5V382|V5V382_9CHRO       IQYSGALENIGFTTREIMKMSVYGLTAA---
tr|B0C9L0|B0C9L0_ACAM1       IQYGEALSNIGFSTGEIMRMSAYGLVAA---
tr|B8HSZ3|B8HSZ3_CYAP4       IQYGEALENIGFSTREIMRMSVYGLSAA---
tr|L8LLH3|L8LLH3_9CHRO       ISYGEALGNIGFNTREIMRLSSQGLKVA---
tr|K9Z5G1|K9Z5G1_CYAAP       ISYGEALANIGFSTREIMKMSSYGLRGA---
tr|I4FNH9|I4FNH9_MICAE       ISYGEALSNIGFSTRDIMRMSAYGLTAV---
tr|I4I7T6|I4I7T6_9CHRO       ISYGEALSNIGFSTRDIMRMSAYGLTAV---
tr|I4HKI6|I4HKI6_MICAE       ISYGEALSNIGFSTRDIMRMSAYGLTAV---
tr|B0JY93|B0JY93_MICAN       ISYGEALSNIGFSTRDIMRMSAYGLTAV---
tr|I4GYG9|I4GYG9_MICAE       ISYGEALSNIGFSTRDIMRMSAYGLTAV---
tr|I4IM50|I4IM50_MICAE       ISYGEALSNIGFSTRDIMRMSAYGLTAV---
tr|I4FD05|I4FD05_MICAE       ISYGEALSNIGFSTRDIMRMSAYGLTAV---
tr|L7E3D3|L7E3D3_MICAE       ISYGEALSNIGFSTRDIMRMSAYGLTAV---
tr|L8NPI8|L8NPI8_MICAE       ISYGEALNNIGFSTRDIMRMSAYGLTAV---
tr|A8YJD6|A8YJD6_MICAE       ISYGEALNNIGFSTRDIMRMSAYGLTAV---
tr|S3JGS6|S3JGS6_MICAE       ISYGEALSNIGFSTRDIMRMSAYGLTAV---
tr|I4GKL8|I4GKL8_MICAE       ISYGEALSNIGFSTRDIMRMSAYGLTAV---
tr|I4HLI6|I4HLI6_MICAE       ISYGEALSNIGFSTRDIMRMSAYGLTAV---
tr|I4FY74|I4FY74_MICAE       ISYGEALSNIGFSTRDIMRMSAYGLTAV---
tr|I4H417|I4H417_MICAE       ISYGEALSNIGFSTRDIMRMSAYGLTAV---
tr|M1LIQ1|M1LIQ1_9SYNC       ISYGEALSNIGFSTREIMRMSSYGLAGV---
tr|H0NYN3|H0NYN3_9SYNC       ISYGEALSNIGFSTREIMRMSSYGLAGV---
tr|H0PB35|H0PB35_9SYNC       ISYGEALSNIGFSTREIMRMSSYGLAGV---
tr|H0PG18|H0PG18_9SYNC       ISYGEALSNIGFSTREIMRMSSYGLAGV---
tr|F7US88|F7US88_SYNYG       ISYGEALSNIGFSTREIMRMSSYGLAGV---
sp|Q55688|ALDEC_SYNY3        ISYGEALSNIGFSTREIMRMSSYGLAGV---
tr|D3EPA5|D3EPA5_ATETH       ISYGEALGNIGFSTREIMRMSSHGLRAS---
tr|B7JUH1|B7JUH1_CYAP8       ISYGEALSNIGFSTRDIMRMSSHGLVAA---
tr|C7QUV0|C7QUV0_CYAP0       ISYGEALSNIGFSTRDIMRMSSHGLVAA---
tr|G5J0P0|G5J0P0_CROWT       ISYGEALGNIGFSTREIMKMSAHGLAAV---
tr|T2JF13|T2JF13_CROWT       ISYGEALGNIGFSTREIMKMSAHGLAAV---
tr|Q4C7T3|Q4C7T3_CROWT       ISYGEALGNIGFSTREIMKMSAHGLAAV---
tr|T2IH71|T2IH71_CROWT       ISYGEALGNIGFSTREIMKMSAHGLAAV---
tr|A3INE1|A3INE1_9CHRO       ISYGEALSNIGFSTREIMKMSSHGLSAA---
tr|B1WR71|B1WR71_CYAA5       ISYGEALSNIGFSTREIMKMSAYGLRAA---
tr|U5DFA3|U5DFA3_9CHRO       VAYSEALSNIGFNAREIARMSVHGLAAA---
tr|L8N0G8|L8N0G8_9CYAN       IQYGESLGKIGFNTRDIMRMSAMGLVAA---
tr|K9SPY2|K9SPY2_9CYAN       IAYGEALGNIGFNTRDIMKMSAMGLLPA---
tr|K9SZC3|K9SZC3_9SYNE       IQYGESLGKIGFNTREIMKMSAMGLVAA---
tr|B4WJ48|B4WJ48_9SYNE       ITYGEALANIGFSARDVMRLSAQGLAAV---
tr|U9WCI1|U9WCI1_9CYAN       ITYGEALSNIGFTSREVMKLSAQGLAMA---
tr|W5U411|W5U411_9CYAN       ITYGEALGNIGFSSRDVMRLSAQGLATA---
tr|K9F0G4|K9F0G4_9CYAN       ITYGEALGNIGFSSRDVMRLSAQGLATA---
tr|K9ULL9|K9ULL9_9CHRO       IAYGEALSNIGFNTGEIMRMSAYGLMGA---
tr|K8GFP8|K8GFP8_9CYAN       IQYGEALSNIGFTTRDVMRLSAMGLSAA---
tr|D4TEY2|D4TEY2_9NOST       IQYGEALSNIGFTTRDIMRLSAYGLATI---
tr|D4TRY1|D4TRY1_9NOST       IQYGEALSNIGFTTRDIMRLSAYGLASI---
tr|K9V615|K9V615_9CYAN       IQYGEALSNIGFNTREIMRLSAYGLRAA---
tr|K9PTP6|K9PTP6_9CYAN       IQYGEALSNIGFTTRDIMRLSAYGLIAA---
tr|K7WTY7|K7WTY7_9NOST       IQYGEALSNIGFTTRDIMRLSAYGLATV---
tr|K9WRL8|K9WRL8_9NOST       IQYGEALSNIGFTTRDIMRLSAYGLTAA---
tr|K9ZJT3|K9ZJT3_ANACC       IQYGEALSNIGFTTRDIMRLSAYGLIPV---
tr|D7E2W1|D7E2W1_NOSA0       IQYGEALSNIGFTTRDIIRLSAYGLATV---
tr|Q112R8|Q112R8_TRIEI       IAYGEALNNIGFTTGEIMRMSAYGLIAA---
sp|Q54764|ALDEC_SYNE7        IAYGEALENIGFTTREIMRMSAYGLAAV---
tr|Q8KPT4|Q8KPT4_SYNE7       IAYGEALENIGFTTREIMRMSAYGLAAV---
tr|Q5N627|Q5N627_SYNP6       IAYGEALENIGFTTREIMRMSAYGLAAV---
tr|K9RFH5|K9RFH5_9CYAN       IQYGEALSNIGFTTRDIMRLSAYGLRAA---
tr|K9TXF6|K9TXF6_9CYAN       IQYGETLANIGFSTRDIMRLSAYGLQAA---
tr|K9X9I4|K9X9I4_9CHRO       IQYGEALSNIGFTTRDIMRMSAYGLTAA---
tr|A0Z9X5|A0Z9X5_NODSP       IQYGETLSNIGFTTRDIMKMSAYGLTAA---
tr|Q3MA38|Q3MA38_ANAVT       IQYGEALSNIGFTTRDIMRMSAYGLTAA---
```

```
                            ...210.......220.......230..
                              :  :*  :*:   :   :   .
tr|Q8YLL5|Q8YLL5_NOSS1      IQYGEALSNIGFTTRDIMRMSAYGLTAA---
tr|K9QXA2|K9QXA2_NOSS7      IQYGEALSNIGFTTRDIMRLSAYGLTAA---
tr|M1X0T0|M1X0T0_9NOST      IQYGEALNNIGFSTRDIMRLSAYGLRKI---
tr|M1WVU9|M1WVU9_9NOST      IQYGEALNNIGFSTRDIMRLSAYGLRKV---
tr|K9S7C1|K9S7C1_9CYAN      IQYGEALDNIGFTTREIMRMSAYGLATI---
tr|K9W4T2|K9W4T2_9CYAN      IAYGEALSNIGFTTRDIMRMSAYGLTAA---
tr|G6FV22|G6FV22_9CYAN      IQYGEALSNIGFNTRDIMRLSAYGLRAA---
tr|W5U379|W5U379_9CYAN      IQYGEALSNIGFNTRDIMRLSAYGLRAA---
tr|W5U417|W5U417_9CYAN      IQYGEALSNIGFNTRDIMRLSAYGLRTA---
tr|K9QA48|K9QA48_9NOSO      IQYGEALSKIGFTTRDIMRLSAYGLSAA---
tr|G9HTG5|G9HTG5_9NOSO      IQYGEALSNIGFTTRDIMRLSAYGLTAA---
tr|H2DDT7|H2DDT7_NOSS6      IQYGEALSNIGFTTRDIMRLSAYGLTAA---
tr|K9YV86|K9YV86_DACSA      IAYGEALSNIGFSTRDIMRMSAYGLREV---
tr|K9Y9U4|K9Y9U4_HALP7      IAYGEALSNIGFNTREIMKMSAYGLREG---
tr|W5U332|W5U332_PLAAG      IAYGEALSNIGFSTRDIMRMSAYGLIAA---
tr|F5UGX9|F5UGX9_9CYAN      IAYGEALGNIGFNNRDIMRMSAQGLAA----
tr|K9VE50|K9VE50_9CYAN      IAYGEALGNIGFNNRDIMRMSAQGLAA----
tr|D8FVZ2|D8FVZ2_9CYAN      IAYGEALSNIGFTTRDIMRMSAYGLTAA---
tr|B4VSW8|B4VSW8_9CYAN      IAYGEALSNIGFTTRDIMRMSAYGLTAA---
tr|K9WII2|K9WII2_9CYAN      IAYGEALSNIGFTTRDIMRMSAYGLTAA---
tr|K9TGS5|K9TGS5_9CYAN      IAYGEALSTIGFTTRDIMRMSAYGLAGA---
tr|B5W0S5|B5W0S5_ARTMA      IAYGEALSNIGFTTRDIMRMSAYGLAGV---
tr|K1WZT6|K1WZT6_ARTPT      IAYGEALSNIGFTTRDIMRMSAYGLAGV---
tr|W6SNU7|W6SNU7_9CYAN      IAYGEALSNIGFTTRDIMRMSAYGLAGV---
tr|H1WAD2|H1WAD2_9CYAN      IAYGEALSNIGFTTRDIMRMSAYGLAGV---
tr|K6DQI0|K6DQI0_ARTPT      IAYGEALSNIGFTTRDIMRMSAYGLAGV---
tr|D5A1Z8|D5A1Z8_ARTPN      IAYGEALSNIGFTTRDIMRMSAYGLAGV---
tr|U7QM55|U7QM55_9CYAN      IAYGEALNNMGFTTREIMRMSAYGLKGA---
tr|A0YKK4|A0YKK4_LYNSP      IAYGEALNNIGFTTREIMRMSAHGLTTA---
tr|A3Z6M0|A3Z6M0_9SYNE      ELFREALESVGFEAVEARRLLMRAAARMV--
tr|B5ILL6|B5ILL6_9CHRO      SSYQEALMDIGFTGREIAKLAAAALVG----
tr|A3YYU8|A3YYU8_9SYNE      TSYQEALTDIGFTTREIAKMATAALLG----
tr|A2C7S0|A2C7S0_PROM3      IAYQESLTEIGFNTREITRMAAAALVS----
sp|Q7V6D4|ALDEC_PROMM       IAYQESLTEIGFNTREITRMAAAALVS----
tr|A5GUD0|A5GUD0_SYNR3      IAYQEALMDIGFNSRDLARMSAAALVA----
tr|K9P3X5|K9P3X5_CYAGP      IAYQDALTDIGFTPREIARMAAAALVG----
tr|Q0I8M9|Q0I8M9_SYNS3      IAYQEALNEIGFSSRDIARMAAAALSI----
tr|G4FIH8|G4FIH8_9SYNE      IAYQEALNEIGFSSRDIARMAAAALTV----
tr|Q05RJ5|Q05RJ5_9SYNE      IAYQEALNEIGFSSRDIARMAAAALAV----
tr|A5GJG5|A5GJG5_SYNPW      IAYQEALEEIGFTSRDIARMAAAALSI----
tr|A4CS34|A4CS34_SYNPV      IAYQEALEQIGFTSRDIARMAAAALAV----
tr|A3Z5H6|A3Z5H6_9SYNE      IAYQEALCEIGFSSRDIARMAAAALAV----
tr|Q060S1|Q060S1_9SYNE      IAYQEALNEIGFSSRDIAKMAAAALAI----
tr|Q3AV61|Q3AV61_SYNS9      IAYQEALNEIGFSSRDIAKMAAAALAI----
tr|Q0QKQ4|Q0QKQ4_9SYNE      IAYNEALSEIGFSSRDIARMAAAALAL----
tr|Q0QM11|Q0QM11_9SYNE      IAYQEALGEIGFTSRDIARMAAAALAV----
tr|Q3ALN2|Q3ALN2_SYNSC      IAYQEALGEIGFTSRDIARMAAAALAV----
tr|W0GVR9|W0GVR9_9SYNE      IAYQEALGEIGFTSRDIARMAAAALAV----
tr|Q0QKF3|Q0QKF3_9SYNE      IAYQEALGEIGFTSRDIARMAAAALAV----
tr|Q7U5G9|Q7U5G9_SYNPX      IAYQEALGEIGFTSRDIARMAAAALAV----
tr|Q0QKK1|Q0QKK1_9SYNE      IAYQEALGEIGFTSRDIARMAAAALAV----
tr|A2C0Z0|A2C0Z0_PROM1      IAYQDSLLEIGLDNREIARMALAAVI-----
tr|Q46GP5|Q46GP5_PROMT      IAYQDSLLEIGLDNREIARMALAAVI-----
tr|A8G3P7|A8G3P7_PROM2      IAYQDALLEMGLDNREIARMAMAAIV-----
tr|A2BVJ4|A2BVJ4_PROM5      IAYQDALLEMGLDNREIARMAMAAIV-----
tr|Q7V2E8|Q7V2E8_PROMP      IAYQDTLLEIGLDNREIARMAMAAIV-----
tr|Q31C02|Q31C02_PROM9      IAYQDTLLEIGLDNREIARMAMAAIV-----
tr|A2BQ13|A2BQ13_PROMS      IAYQDTLMEIGLDNREIARMAMAAIV-----
tr|A3PBQ6|A3PBQ6_PROM0      IAYQDTLMEIGLDNREIARMAMAAIV-----
tr|A9BEF6|A9BEF6_PROM4      IAYQDSLMEIGLDAREIARMALAAIA-----
tr|Q7VD51|Q7VD51_PROMA      IAYQDSLLEIGLDSREIARMALAALV-----
tr|B8HLW2|B8HLW2_CYAP4      TRYGEALGQIGFKLSEILSLSVQGLQAVTP-
tr|U9VZT6|U9VZT6_9CYAN      VNYSEALKEIGFNTREILRMASHGLTSSDQR
tr|K9EQR0|K9EQR0_9CYAN      VHYSEALKEIGFSSREILRMASHGLASSKQR
```
