# Supplementary figures and images for: Role of Cysteine Residues in the Structure, Stability, and Alkane Producing Activity of Cyanobacterial Aldehyde Deformylating Oxygenase
Source: PLoS One. 2015 Apr 2;10(4):e0122217. doi: 10.1371/journal.pone.0122217 (PMC4383598; doi:10.1371/journal.pone.0122217)

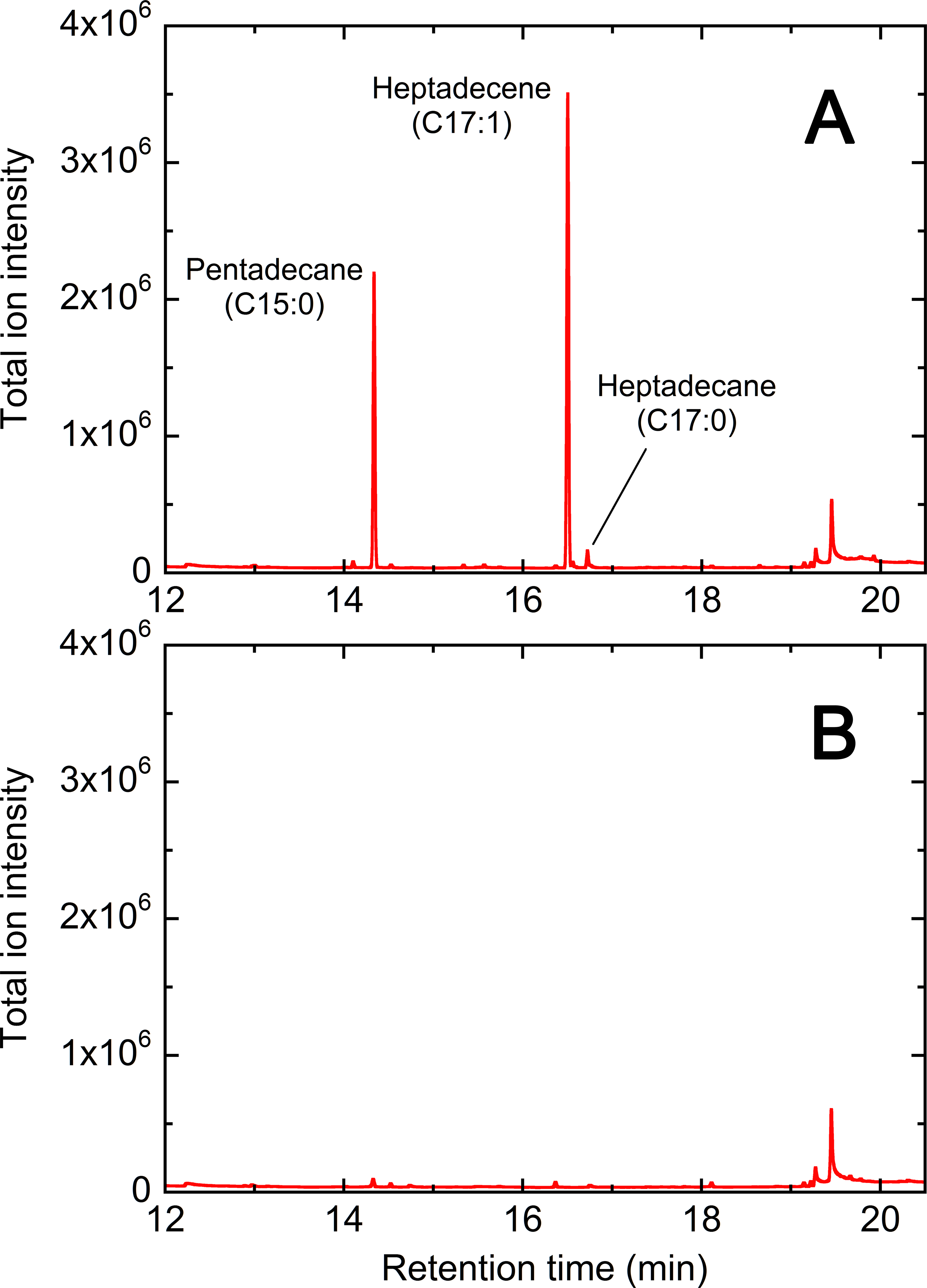

Supplement: S2 Fig — (A) GC-MS profile of the E. coli cell culture coexpressing wild type AD and AAR proteins. The peaks after 19 min of the elution time were derived from E. coli cells. (B) GC-MS profile of the E. coli cell culture that did not express either AAR or AD (control). Peaks for pentadecane, heptadecene, and heptadecane, which should appear between 14–17 min, were not observed. (TIF) [file pone.0122217.s002.tif]

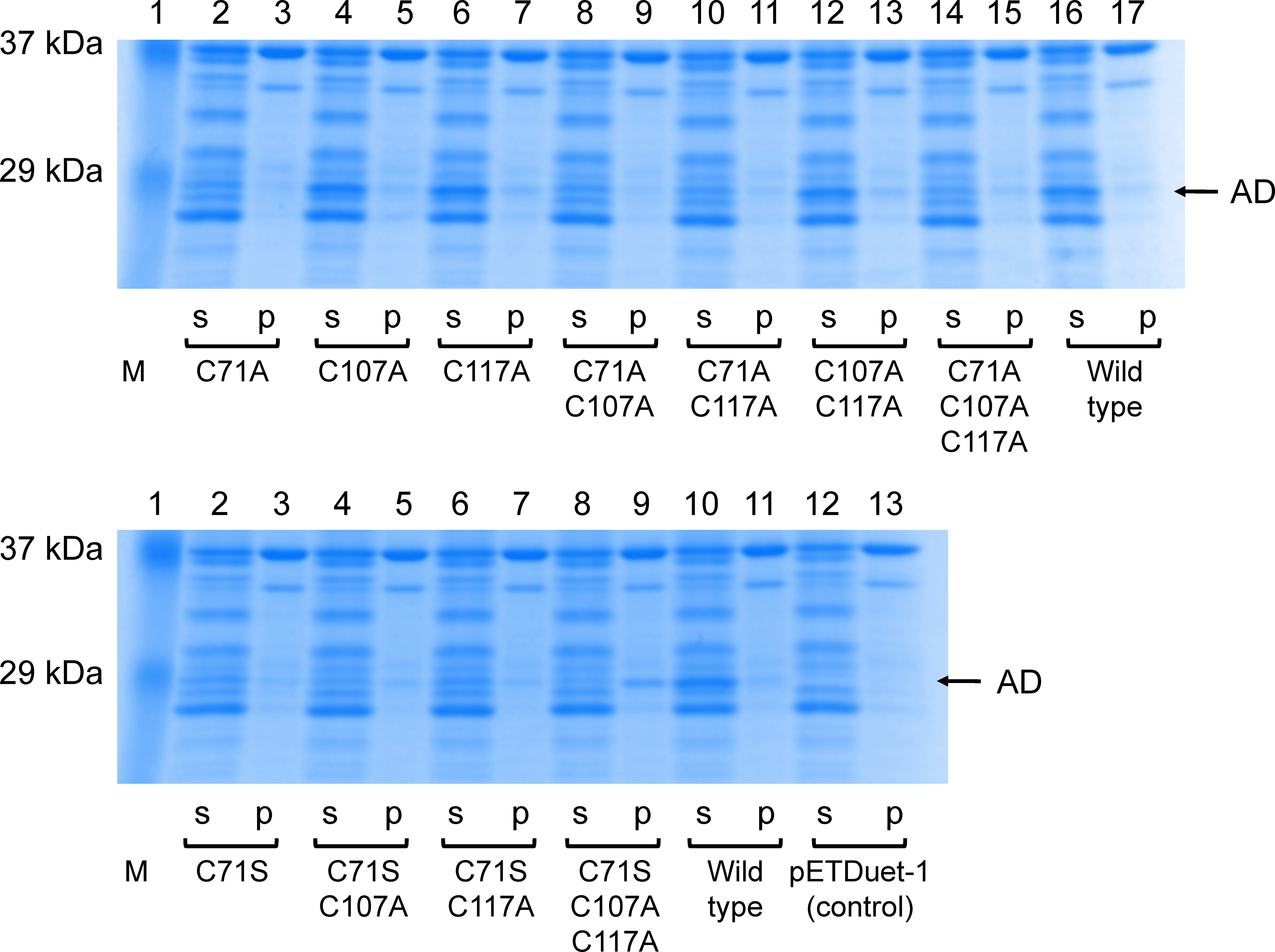

Supplement: S3 Fig — The cell culture was sonicated and centrifuged to separate the supernatant (s) and pellet (p) fractions. Lane 1 is the molecular weight marker (M). The band for AD (28 kDa) is indicated by an arrow. (TIF) [file pone.0122217.s003.tif]

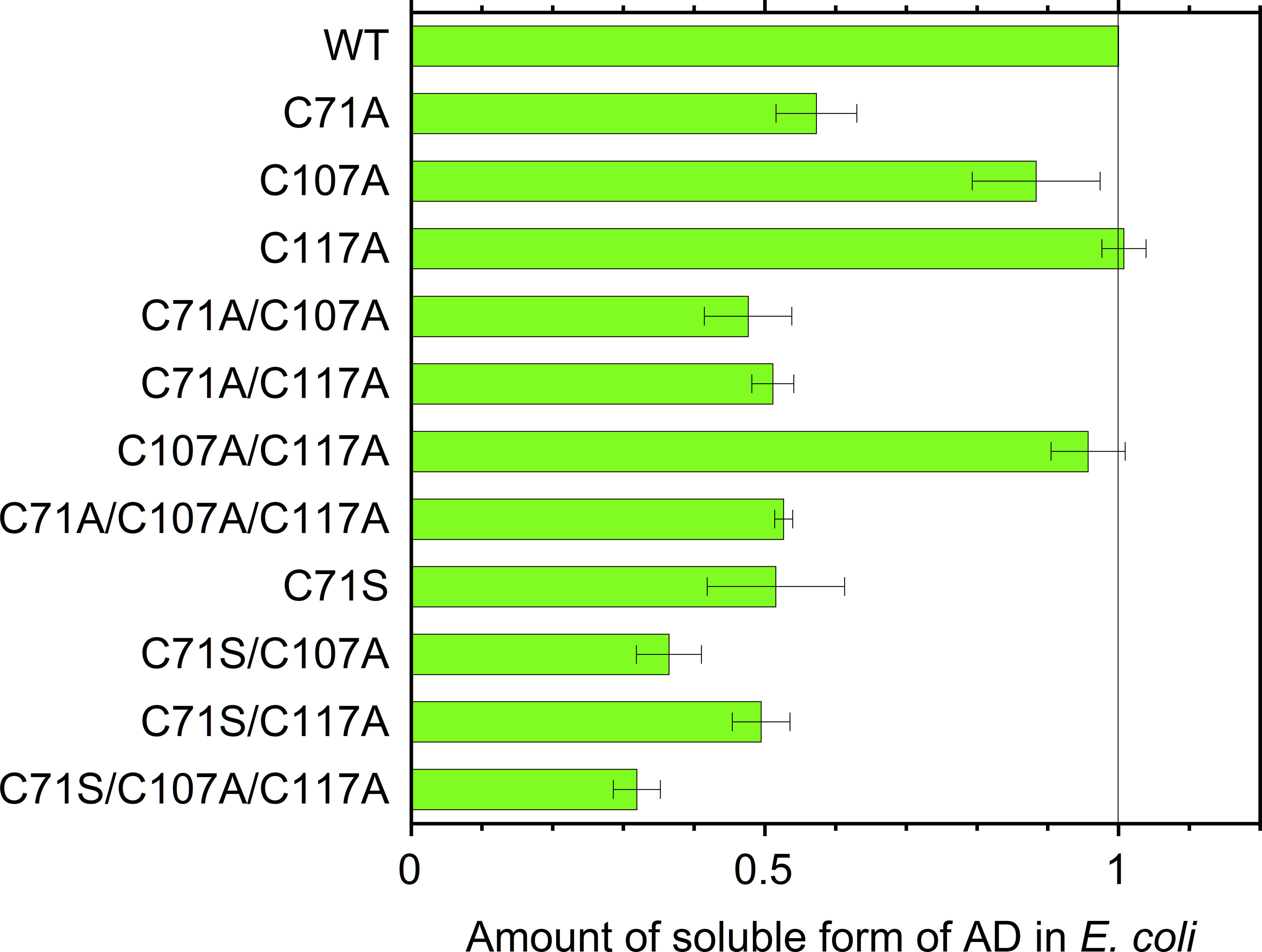

Supplement: S4 Fig — The value was quantified by SDS-PAGE. Values relative to that of the wild type are shown. The data are means ± standard deviations of duplicate or quadruplicate experiments. (TIF) [file pone.0122217.s004.tif]

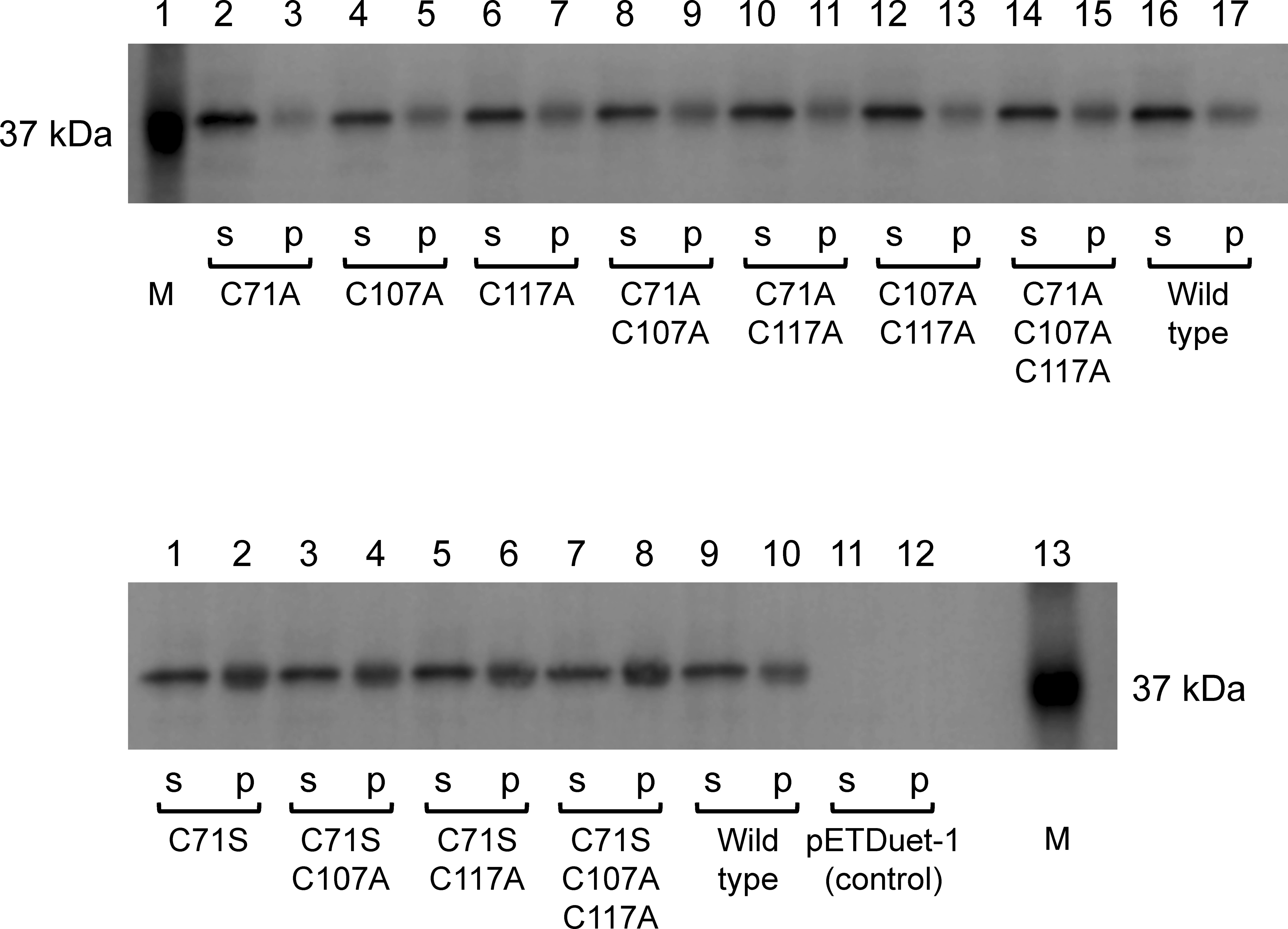

Supplement: S5 Fig — The cell culture was sonicated and centrifuged to separate the supernatant (s) and pellet (p) fractions. Lanes 1 and 13 in the upper and lower gels, respectively, are the molecular weight marker (M). (TIF) [file pone.0122217.s005.tif]

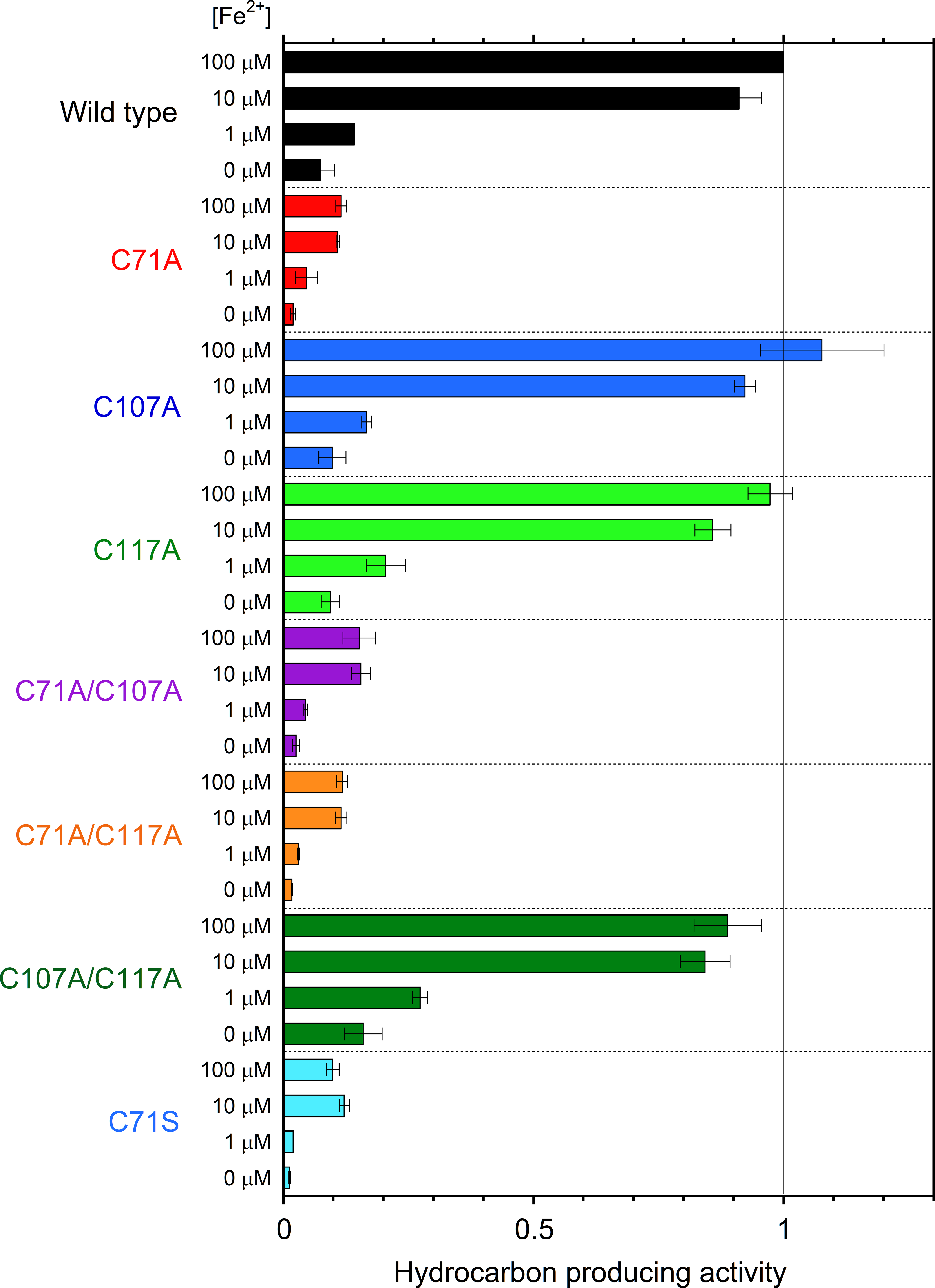

Supplement: S6 Fig — The amount of hydrocarbons produced in E. coli, which was cultured in the M9 medium containing 0, 1, 10, or 100 μM ammonium iron (II) sulfate, was normalized by the amount of soluble form of AD protein in E. coli, as estimated by SDS-PAGE. The activity value presented here is relative to that of the wild type in the presence of 100 μM ammonium iron (II) sulfate. The data are means ± standard deviations of duplicate or quadruplicate experiments. (TIF) [file pone.0122217.s006.tif]

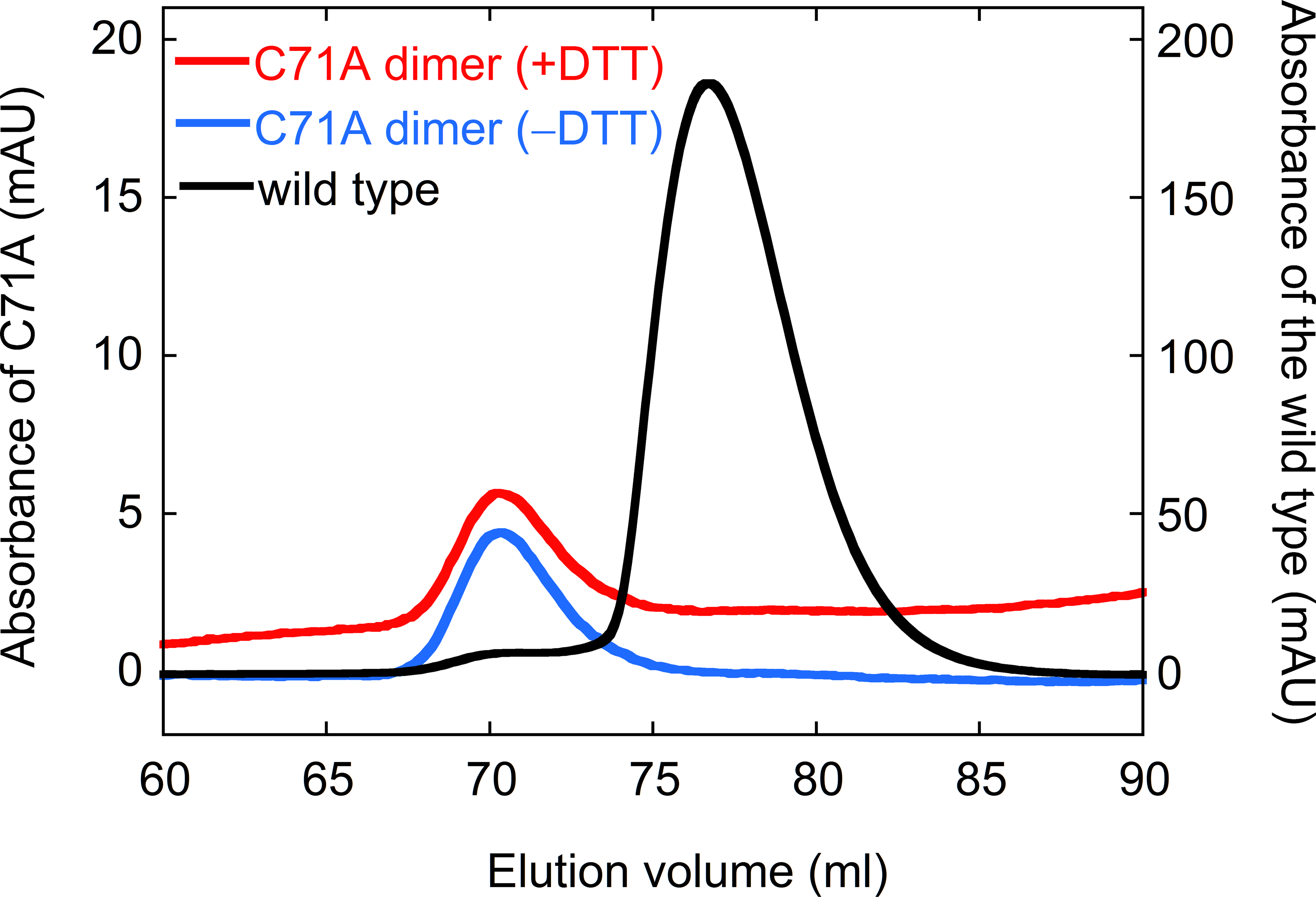

Supplement: S7 Fig — (Red) The dimer fraction of C71A was purified by size exclusion chromatography, concentrated with an Amicon Ultra-4 centrifugal filter, treated with 100 mM DTT at 4°C for 3 h, and subjected to size exclusion chromatography with running buffer containing 5 mM DTT. (Blue) The elution profile of the purified and concentrated dimer fraction of C71A applied to size exclusion chromatography using running buffer without DTT. (Black) The elution profile of wild type AD is shown as a control. The absorbance scales for C71A and wild type AD are shown at the left and right vertical axes, respectively. Here, a shorter column is used compared to that used in the experiments shown in Fig 3. (TIF) [file pone.0122217.s007.tif]

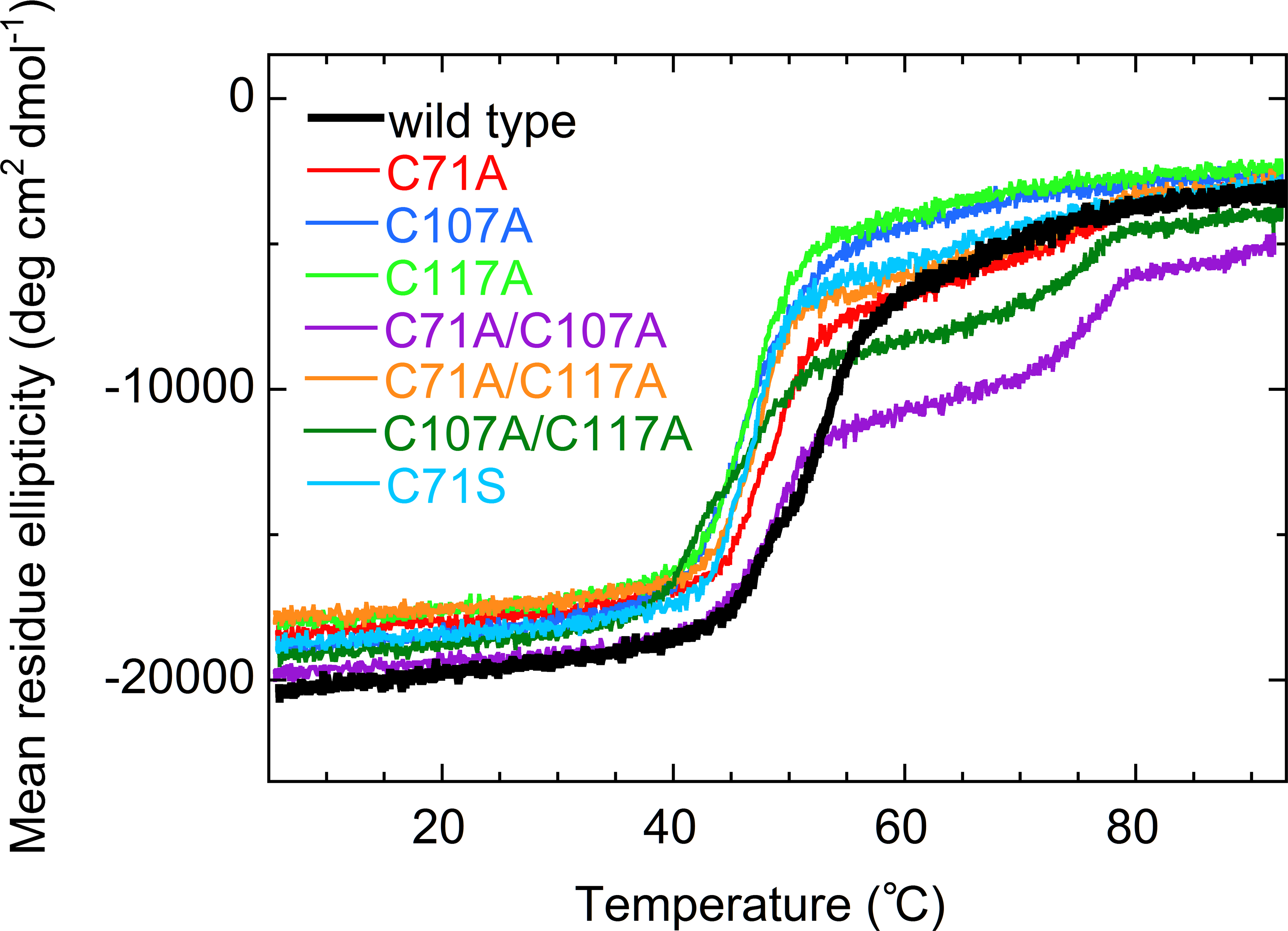

Supplement: S8 Fig — (TIF) [file pone.0122217.s008.tif]
